# Dihydrotestosterone: Biochemistry, Physiology, and Clinical Implications of Elevated Blood Levels

**DOI:** 10.1210/er.2016-1067

**Published:** 2017-05-02

**Authors:** Ronald S. Swerdloff, Robert E. Dudley, Stephanie T. Page, Christina Wang, Wael A. Salameh

**Affiliations:** 1 Division of Endocrinology, Department of Medicine, David Geffen School of Medicine at UCLA, Torrance, California 90502; 2 Clarus Therapeutics, Inc., Northbrook, Illinois 60062; 3 Division of Metabolism, Endocrinology, and Nutrition, University of Washington School of Medicine, Seattle, Washington 98195; 4 UCLA Clinical and Translational Science Institute, Harbor-UCLA Medical Center, and Los Angeles Biomedical Research Institute, David Geffen School of Medicine at UCLA, Torrance, California 90509

## Abstract

Benefits associated with lowered serum DHT levels after 5*α*-reductase inhibitor (5AR-I) therapy in men have contributed to a misconception that circulating DHT levels are an important stimulus for androgenic action in target tissues (*e.g.*, prostate). Yet evidence from clinical studies indicates that intracellular concentrations of androgens (particularly in androgen-sensitive tissues) are essentially independent of circulating levels. To assess the clinical significance of modest elevations in serum DHT and the DHT/testosterone (T) ratio observed in response to common T replacement therapy, a comprehensive review of the published literature was performed to identify relevant data. Although the primary focus of this review is about DHT in men, we also provide a brief overview of DHT in women. The available published data are limited by the lack of large, well-controlled studies of long duration that are sufficiently powered to expose subtle safety signals. Nonetheless, the preponderance of available clinical data indicates that modest elevations in circulating levels of DHT in response to androgen therapy should not be of concern in clinical practice. Elevated DHT has not been associated with increased risk of prostate disease (*e.g.*, cancer or benign hyperplasia) nor does it appear to have any systemic effects on cardiovascular disease safety parameters (including increased risk of polycythemia) beyond those commonly observed with available T preparations. Well-controlled, long-term studies of transdermal DHT preparations have failed to identify safety signals unique to markedly elevated circulating DHT concentrations or signals materially different from T.

## Teaser

Circulating levels of DHT in response to testosterone replacement therapy (TRT) do not correlate with those found in androgen sensitive tissue due to homeostatic control of intracellular DHT.

**Essential Points**

- Circulating levels of DHT in response to testosterone replacement therapy (TRT) do not correlate with those found in androgen sensitive tissue (e.g., prostate, adipose, muscle) due to local regulatory mechanisms that tightly control intracellular androgen homeostasis.
- The modest increases observed in serum DHT and in the DHT/T ratio observed after TRT are unlikely to be a cause of clinical concern, particularly when viewed in the context of changes observed in these parameters for currently marketed T-replacement products and those under development for which DHT data are available.
- While well-controlled, long-term studies designed to specifically examine the effects of androgen exposure on risk for prostate need to be conducted, the current clinical data base is relatively reassuring that circulating levels of androgens (or changes in such) apparently do not play as pivotal a role as once thought in the development of prostate disease.
- Robust epidemiologic or clinical trial evidence of a deleterious DHT effect on CVD is lacking. There is some evidence that DHT therapy in men with CVD may improve clinical status—a finding that needs confirmation. Data from a longitudinal data base of older normal (i.e., not hypogonadal) indicated an association between serum DHT and incident CV disease and mortality. Conversely, others have reported that higher DHT levels in older men were associated with decreased all-cause mortality and reduced ischemic heart disease mortality. Additional exploration in prospective, placebo-controlled intervention studies of TRT with CVD as the primary endpoint is needed to resolve the long-term effects of androgens on CVD risks.
- DHT does not play a substantive role in body composition compared to T under normal conditions. Thus, elevated levels of DHT in response to TRT are unlikely to appreciably impact lean or fat mass. Nonetheless, data from animals suggest a role for DHT in adipose tissue that inhibits biochemical pathways involved in lipid synthesis and promotes several transcripts associated with apoptosis of adipocytes. Whether these DHT-induced effects also occur in human adipose tissue remains an area for future study.
- There is very limited data available regarding DHT and effects on cognition. Further research is needed, particularly in light of animal data where DHT positively modified synaptic structure and significantly delayed cognitive impairment in a well-regarded animal model for Alzheimer’s disease.
- Recent data indicating that higher levels of DHT were inversely associated with insulin resistance and risk of diabetes merit further mechanistic investigation to understand whether this action is separate from that of T.

This review on dihydrotestosterone (DHT) biology and the clinical implications of serum DHT concentrations clarifies concepts that are of importance in clinical practice.

DHT is the 5*α*-reduced metabolite of testosterone (T) that is principally converted from T in target organs such as prostate, skin, and liver. Synthesis can also occur from other precursors, but these pathways, although potentially important in some tissues (*e.g.*, in prostate), are minor. Intracellular DHT is a more potent androgenic agonist than T, and its presence in some tissues such as the prostate is necessary for the full organ development and function. Circulating DHT levels are of much less importance than T for optimizing the intracellular DHT concentrations due to the presence of a rate-limiting enzyme, 5*α*-reductase (SRD5A; types I and II). Inhibition of these enzymes with 5*α*-reductase inhibitors (5AR-Is) decreases intratissue DHT levels and thus, in certain tissues (*i.e.*, prostate), diminishes the agonist action of T, thus reducing prostate size and function. These inhibitors have been used to reduce prostate hypertrophy and the symptoms of lower urinary tract obstruction in benign prostate hypertrophy (BPH). 5AR-Is have been associated with reduced risk of prostate cancer, but they have not been approved for this purpose (1–3). Suppression of intracellular DHT levels with 5AR-Is results in reduced levels of DHT in the blood due to reduced leakage of DHT from peripheral target organs and reduced conversion of T to DHT from Leydig cells in the testes.

The clinical benefits associated with lowered serum DHT levels after 5AR-Is appear to have led to the misconception that circulating DHT is an important stimulus for androgenic action in the prostate gland. However, studies in which serum DHT concentrations were markedly elevated by exogenous administration of DHT had almost no effect on prostate DHT concentrations, prostate size, and lower urinary tract symptoms (see “Intraprostatic Control Of DHT in the Presence of Fluctuating Levels of Circulating Androgens” and associated references). The reason for this highlights fundamentally important control mechanisms in androgen target tissues that finely regulate pathways for androgen synthesis and degradation to maintain DHT homeostasis. These intracellular processes do not appear to be affected by circulating DHT concentrations. Furthermore, it is well documented that DHT can be synthesized in androgen-sensitive tissues such as prostate from substrates other than T (*e.g.*, from 17-hydroxypregnenolone and 17-hydroxyprogesterone in what is termed the “backdoor” pathway and from 5*α*-androstane-3*α*, 17-*β*-diol via the intracrine reverse synthesis pathway) (4). We will also explore the implications of modest increases in serum DHT that are seen with T replacement therapy (TRT; including, for completeness, DHT preparations) for male hypogonadism and discuss why these likely have minimal clinical implications for men treated with androgens.

Serum DHT levels are dependent upon the concentration of serum T achieved with TRT and the expression of normal levels of functional SRD5A in tissues. In adult eugonadal men, serum DHT levels are about one-tenth that of total serum T concentrations. As would be expected, the pattern of rise in DHT generally tracks with the increase in T, but the magnitude of change is substantially less. Differences in circulating DHT in response to various routes of T and prodrug (*e.g.*, T esters) administration have been reported. In some cases, this can result in supraphysiologic DHT concentrations, thus leading to an important clinical question: What are the potential health effects of supraphysiological serum DHT concentrations in the setting of androgen therapy (*e.g.*, TRT)?

To assess the clinical significance of modest elevations in serum DHT and DHT/T ratio observed with some delivery systems of TRT, we performed a comprehensive review of the published literature to identify relevant data. We examined not only studies in which elevated DHT was documented, but also those where 5AR-Is were used to suppress DHT production. Where appropriate, we have also included data from salient animal studies, although the focus of our analyses is principally on human data. In the case of some currently available TRT preparations, no pertinent published DHT data were available, and thus they are not included in this review. This points to a weakness in some studies of TRT or SRD5A inhibition, namely, the absence of data on circulating DHT levels. A notable case in this regard is the Prostate Cancer Prevention Trial (1), which evaluated the effects of 5AR-I treatment but did not directly measure serum DHT in the men treated with finasteride. Instead, serum 5*α*-androstane-3*α*, 17*β*-diol glucuronide, a distal metabolite of DHT, was used as a surrogate measure of intraprostatic DHT (5).

Our review is focused primarily on DHT actions in men given historical concern about potential adverse effects of elevated DHT on prostate. However, for completeness, we have included additional potential tissue targets of DHT as well a brief section summarizing what is known regarding DHT in women.

## Overview of DHT Biochemistry/Physiology

### Endogenous formations and localization

**Figure 1.**
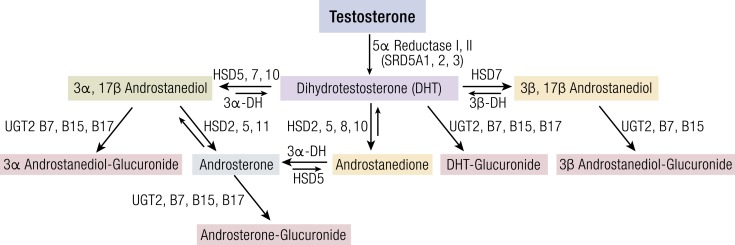
Metabolism pathways for deactivation of DHT to inactive glucuronides. Enzymes and genes associated with pathways are noted next to arrows. Relative thickness/size of arrows represents primary direction of reaction. Compiled from (6, 7, 8). 3*α*-DH, 3*α*-dehydrogenase; 3*β*-DH, 3*β*-dehydrogenase; HSD2, 17*β*-hydroxysteroid dehydrogenase type 2; HSD3, 17*β*-hydroxysteroid dehydrogenase type 3; HSD5, 17*β*-hydroxysteroid dehydrogenase type 5; HSD7, 17*β*-hydroxysteroid dehydrogenase type 7; HSD8, 17*β*-hydroxysteroid dehydrogenase type 8; HSD10, 17*β*-hydroxysteroid dehydrogenase type 10; HSD11, 17*β*-hydroxysteroid dehydrogenase type 11; SRD5A1, 5*α*-reductase type 1; SRD5A2, 5*α*-reductase type 2 (the 5α-reductase gene that predominates in androgen-sensitive tissue); SRD5A3, 5*α*-reductase type 3; UGT2, B7, UDP-glucuronyltransferase type 2 isozyme B7; UGT2, B15, UDP-glucuronyltransferase type 2 isozyme B15; UGT2, B17, UDP-glucuronyltransferase type 2 isozyme B17.

DHT is one of four principle androgens in humans and is synthesized primarily via the irreversible action of microsomal SRD5A (both types I and II) on T (Fig. 1). This saturable process follows Michaelis-Menton kinetics and is not affected by age (9). Localization of SRD5As in prostate tissue (type II), skin (type I), liver (types I and II), and hair follicles (primarily type I) catalyzes the formation of DHT from T in these tissues. These enzymes (expressed in the nucleus and cytoplasm of, for example, prostate epithelial cells) (10) are encoded by the 5*α*-reductase type 2 (SRD5A2) gene, and polymorphisms of this gene (leading to increased 5*α*-reductase activity and DHT concentrations in prostate) have been hypothesized to increase risk of prostate cancer (11). The SRD5A3 gene has also been linked to increased DHT production in hormone refractory prostate cancer cells (12), and this gene may be particularly important in metastatic prostate cells, which have been shown to express more SRD5A1 and SRD5A3 but significantly less SRD5A2 (13). Conversion of T to DHT via SRD5A activity in peripheral tissue is the main source of circulating DHT (14, 15), but it is important to note that little DHT synthesized in the prostate or liver enters the general circulation due to efficient intracellular mechanisms that initially metabolize DHT to 3*α*- and 3*β*-, 17*β*-androstanediol that have little androgen activity (16, 17). As noted previously, DHT can also be synthesized in tissues by “backdoor pathways” that enable formation of DHT in the absence of T or androstenedione as precursors (18, 19). In yet a third synthetic pathway to DHT, namely, the 5*α*-androstanedione pathway, 5*α*-androstanedione is converted by 17*β*-hydroxysteroid B3 to DHT (11). As discussed later, these alternate synthetic pathways, which are not influenced by circulating DHT, may have particular clinical significance within prostate tissue.

### Binding affinity for AR

Binding of T and DHT to the androgen receptor (AR) stabilizes the AR and slows what would otherwise be rapid degradation. At low circulating androgen levels, DHT binding is favored over T but at higher relative T concentrations (*e.g.*, eugonadal state), stabilization of the AR is driven by T more than DHT (20). Nonetheless, DHT is the most potent endogenous androgen based on four critical aspects of its binding to the AR. First, DHT has a relative binding affinity for the AR that is roughly 4 times that of T (21). Second, the rate of dissociation from the AR is about 3 times slower than T (22). Third, binding of DHT to the AR transforms the AR to its DNA-binding state (23). And lastly, DHT upregulates AR synthesis and reduces AR turnover (24). Collectively, these processes amplify the androgenic action of DHT and increase its potency compared with T. However, this may lead to the incorrect conclusion that binding of DHT to the AR always occurs preferentially over T. This is too simplistic a view and ignores the importance of intracellular control of T and DHT concentrations that are mediated by a host of local metabolic pathways. Organ differences in receptor binding of T and DHT result, in part, from relative differences in intracellular concentrations of these androgens rather than from differences in receptor affinities alone (22). Indeed, it has been clearly demonstrated that high concentrations of intracellular T can shift AR binding away from DHT by mass action (25). Moreover, despite there being a single AR, physiological differences in T and DHT action are well known and likely reflect variations in AR receptor distribution, ligand-induced conformational changes to AR that effect stabilization, local hormone synthesis and metabolism, AR-ligand interactions with chromatin, cooperativity of receptors with other transcription factors, and actions of coactivators and corepressors (26, 27). Thus, local tissue control of androgen levels in conjunction with numerous other factors drive AR-induced transcriptional responses. And as elucidated later in this review, tissue concentrations of androgens (particularly in the prostate) are partly distinct from circulating levels.

### Protein binding

Like T, circulating DHT is principally bound to sex hormone–binding globulin (SHBG) and, more weakly, to albumin. In general, protein-bound DHT is inactive except in some reproductive tissues in which megalin, an endocytic receptor, acts as a pathway for cellular uptake of DHT when bound to SHBG (28). Studies of interactions between a wide array of natural and synthetic androgens and SHBG indicate that the molecular structure of DHT favors tight linkage to the steroid binding site on SHBG (29). Compared with T, DHT has roughly a fivefold greater binding affinity to SHBG (30). Binding of circulating DHT to SHBG is highest in young males 0.5 to 2 years of age (90%) and thereafter declines to about 70% at age 15 and to 40% in young adult men (age 18) (31). The increase of SHBG that occurs with aging (approximately 1% per year) increases DHT binding in older men (32–35). Dissociation rate constants from SHBG for DHT and T have been measured in human serum and correspond to half times of dissociation of 43 (DHT) and 12 (T) seconds, thus further demonstrating the tenacity to which DHT binds to SHBG (36). Accordingly, concentrations of free circulating DHT in eugonadal men are very low and would be expected to remain so even when total DHT levels increase in response to TRT.

This leads to an important question: Can an increase in circulating levels of SHBG-DHT give rise to DHT-mediated effects? It is well known that SHBG can bind to cell membranes and interact with the SHBG receptor (R_SHBG_), thus potentially providing a means for its bound ligand to enter the cell. In the case of SHBG-DHT, studies have shown that this complex does not bind to the R_SHBG_ (37). However, once formed, the SHBG-R_SHBG_ can be activated by an agonist steroid to initiate downstream events beginning with the activation of adenylyl cyclase and the generation of cyclic adenosine monophosphate (cAMP) (37). Generation of cAMP in this scenario has been shown to be steroid specific. For example, when DHT or estradiol were exposed to unbound SHBG in a human prostate cancer cell line (namely, LNCaP), rapid increases in intracellular cAMP were observed. However, when this experiment was conducted with human prostatic explants, estradiol caused a rise in cAMP but DHT did not (37).

### Metabolism

DHT formed in peripheral tissues is extensively metabolized before its metabolites appear in the circulation (38, 39). Metabolism of DHT to inactive steroids occurs primarily via the initial actions of 3*α*-17*β*-hydroxysteroid dehydrogenase (3*α*-HSD) and 3*β*-17*β*-hydroxysteroid dehydrogenase (3*β*-HSD) in liver, intestine, skin, and androgen-sensitive tissues. Subsequent conjugation by uridine 5′-diphospho (UDP)-glucuronyltransferase (UGT) is the major pathway for urinary and biliary elimination of DHT metabolites and, locally, is the principal irreversible step to protect tissues from high concentrations of this potent androgen (Fig. 1). Of the UGTs, only UGT2 isozymes participate in DHT metabolism. In this regard, UGT2B7, B15, and B17 have remarkable capacities to conjugate androgens and are abundant in androgen-sensitive tissues (6). Differential expression of UGT2 isozymes has been reported and likely plays a role in tissue DHT concentrations independent of circulating androgen levels, particularly in androgen-sensitive tissue. For example, transcripts of UGT2B7, B15, and B17 have been identified in liver, intestine, skin, breast, uterus, and ovary, but adipose tissue expresses only UGT2B15, whereas in prostate, UGT2B15 and B17 are expressed only in luminal and basal cells, respectively. This differential localization combined with other local differences in androgen-metabolizing enzymes provides a finely tuned mechanism for control of intracellular androgen concentrations (7). Polymorphisms of UGT2B15 (that is highly effective in conjugating DHT and its metabolites) have been identified (40) and are postulated to protect prostate tissue from high DHT concentrations and thus lower prostate cancer risk (41, 42). Conversely, increased prostate cancer risk had been observed in white but not African American men with UGT2B17 deletion polymorphism (43). So although it is generally true that DHT concentration in tissue is finely regulated (and, as discussed later, probably not effected to any relevant degree by circulating levels observed in response to androgen therapy), it is equally true that polymorphisms in genes responsible for androgen metabolism may perturb this homeostatic mechanism, thus leading to clinically relevant consequences—both positive and negative.

Finally, the metabolism of DHT must also be considered in light of its metabolic clearance. The overall metabolic clearance of DHT and its metabolism in muscle and adipose tissue of normal men were evaluated in response to intravenously infused DHT (15, 44). The overall mean metabolic clearance of DHT was roughly 70% that of T, thus indicating a modestly longer residence time for DHT. Metabolism of DHT was substantially greater in adipose tissue compared with T, and there was little conversion of T to DHT in muscle. Metabolism of intravenously administered DHT compared with transdermally applied DHT revealed that skin is a major site of peripheral DHT metabolism to 3*α*-androstanediol, whereas intravenously-administered DHT yielded greater concentrations of 3*α*-androstanediol-glucuronide (45). Splanchnic tissues have a high capacity to metabolize DHT to DHT-glucuronide, which has importance when oral androgens like T undecanoate (TU) are administered (46). A large fraction of DHT produced in the liver is metabolized to DHT-glucuronide prior to subsequent entry into the circulation (17).

### Analytical methods for DHT quantification

In adult eugonadal men, serum DHT concentrations are most accurately measured by liquid chromatography tandem-mass spectrometry (LC-MS/MS), and consistent normal ranges based on this assay platform have been reported across several studies of men spanning a wide age range. A DHT reference range of 14 to 77 ng/dL (0.47 to 2.65 nmol/L) for healthy adult men (18 to 59 years; n = 113) has been reported by Shiraishi *et al.* (47). Handelsman *et al.* (48) evaluated age-specific population profiles of circulating DHT in community-dwelling men (<65 years; n = 2606) and observed a serum DHT range of 23 to 102 ng/dL (0.8 to 35 nmol/L). In a cohort of healthy older men (71 to 87 years; n = 394), a DHT reference range of 14 to 92 ng/dL (0.49 to 3.2 nmol/L) has been reported (49). Finally, a normal DHT range of 11 to 95 ng/dL (0.38 to 3.27 nmol/L) has been published by a well-regarded commercial clinical laboratory that utilizes LC-MS/MS for the assay of DHT (Mayo Clinical Medical Laboratory, Rochester, MN). In eugonadal men, DHT concentrations are roughly 7- to 10-fold lower than circulating concentrations of T. Also of note is that plasma T and DHT tend to be highly correlated with a correlation coefficient of 0.7 (49).

Prior to the advent of LC-MS/MS for measurement of DHT, less-precise direct DHT immunoassay methods were used in older studies [*e.g.*, direct radioimmunoassay (RIA), enzyme-linked immunosorbent assay (ELISA)]. We now know that these older assays yielded consistently higher T and DHT values compared with LC-MS/MS by up to 25% (50), particularly at low hormone levels. Others have reported that serum DHT measured by RIA overestimated DHT based on LC-MS/MS by as much as 40% (47). These discrepancies are likely due to lack of specificity of the DHT antibody used in the RIA and failure to remove T from the assay that contributes to cross-reactivity. Because of this, some caution must be exercised in the interpretation of DHT values not measured by LC-MS/MS or by RIA in the absence of Celite column chromatography or other methods to remove T prior to DHT immunoassay. However, when DHT is administered exogenously in pharmacologic amounts, circulating DHT levels increase dramatically, whereas there is a parallel drop in luteinizing hormone and T. Consequently, the use of older RIA methods in situations where DHT levels were high likely yielded reasonably accurate measures of DHT and DHT/T ratios because the mass excess of DHT would have minimized the impact of cross-reactivity with T. In this review, we have noted how T and DHT were measured in each of the studies considered. Findings from studies in which DHT and DHT/T ratios were reported based on LC-MS/MS are more informative and should be afforded more weight.

## Serum DHT and DHT/T Ratios in Men After Transdermal DHT Administration

Data regarding the clinical impact of sustained supraphysiologic concentrations of DHT in men repeatedly exposed to daily transdermally administered DHT gel provide valuable clinical safety information. Here we summarize the findings from three placebo-controlled studies in which men were treated with a transdermal DHT gel formulation for 3, 6, or 24 months.

### Transdermal DHT gel in older men with partial androgen deficiency treated for 3 and 6 months

The efficacy and safety of a transdermal DHT gel was studied by Ly *et al.* (51) and Kunelius *et al.* (52) in placebo-controlled studies in older men with partial androgen deficiency who were treated for 3 and 6 months, respectively. Table 1 summarizes the effect of DHT treatment on serum T, DHT, and DHT/T ratio in response to DHT gel. T and DHT concentrations and DHT/T ratios remained stable in the placebo gel group. As would be expected, serum T concentrations in men treated with DHT gel were significantly suppressed to about one-third of baseline whereas serum DHT concentrations increased dramatically, rising about 10-fold. In parallel, the DHT/T ratio increased about 16- to 40-fold across the two studies. Despite such high serum DHT levels, DHT gel treatment did not significantly increase total, central, or peripheral prostate volumes, as measured by ultrasonography, nor was serum prostate-specific antigen (PSA) elevated. In addition, International Prostate Symptom Scores (IPSS) remained unchanged in men treated with DHT gel for 6 months. Exogenous DHT therapy was associated with a modest increase in hematocrit (without exceeding the normal upper limit) but was without effect on serum lipids or other parameters of cardiovascular (CV) risk.

**Table 1.** **Effect of DHT Treatment on Mean (± Standard Deviation) Serum T and DHT Concentrations and Prostate and CV Risk Factors**

Study Description and Population	Duration (Months)	N (Completed)	T (ng/dL) [nmol/L]	DHT (ng/dL) [nmol/L]	DHT/T*^c^*	Assay Method	Effect of DHT on Prostate and CV Risk Factors
Daily application of DHT gel (70 mg/d)	3	17*^a^*, DHT gel	Baseline: 432 ± 89 [14.98 ± 3.09]	Baseline: 41 ± 12 [1.41 ± 0.41]	0.09	RIA	Increase in Hgb/HCT but remained in normal range
Older men; age, >60; T <450 ng/dL (51, 53)			1 mo: 210 ± 14 [7.28 ± 0.49]	1 mo: 490 ± 58 [16.87 ± 2.0]	2.44		HDL cholesterol did not change
			2 mo: 187 ± 14 [6.48 ± 0.49]	2 mo: 505 ± 58 [17.39 ± 2.0]	2.70		No evidence of stimulatory effects on prostate volume or PSA concentrations
			3 mo: 144 ± 57 [4.99 ± 1.98]	3 mo: 534 ± 99 [18.39 ± 3.41]	3.71		No impairment in brachial artery size or flow in response to glyceryl trinitrate–induced dilatation
							No change in inflammatory biomarkers (CRP, sVCAM, and sICAM)
Daily application of DHT gel (125–250 mg/d)	6	54*^b^*, DHT gel	Baseline: 464 ± 132 [16.26 ± 4.58]	Baseline: 44 ± 17 [1.51 ± 0.59]	Baseline: 0.09	RIA	No effect on serum lipids
				3 mo: 270 ± 136 [9.36 ± 4.68]			
			6 mo: 170 ± 112 [5.89 ± 3.88]	6 mo: 238 ± 133 [8.19 ± 4.58]	6 months: 1.4		
		14, DHT gel (125 mg/d)		Baseline: 44 ± 20 [1.51 ± 0.69]			
				3 mo: 276 ± 200 [9.50 ± 6.89]			Increase in HCT (2.3%) and Hgb (0.9 g/L) at 6 months
				6 mo: 247 ± 189 [8.50 ± 6.51]			
Older men; mean age, 58 (52)		27, DHT gel (187.5 mg/d)		Baseline: 44 ± 17 [1.51 ± 0.59]			
				3 mo: 261 ± 113 [8.99 ± 3.89]			
				6 mo: 238 ± 139 [8.19 ± 4.79]			Serum PSA, prostate volume, and IPSS remained unchanged
		19, DHT gel (250 mg/d)		Baseline: 44 ± 20 [1.51 ± 0.69]			
				3 mo: 267 ± 119 [9.19 ± 4.10]			
				6 mo: 232 ± 81 [7.99 ± 2.79]			
Daily application of DHT gel (70 mg/d)	24	37, DHT gel	Baseline: 493 ± 176 [17.1 ± 6.1]	Baseline: 64 ± 61 [2.2 ± 2.1]	Baseline: 0.13	LC-MS/MS	No effect on lipids
							No effect on carotid IMT
							Decreased (–1.1 kg) fat mass by DEXA
Healthy men older than 50 years with no known prostate disease (54)							Increased HCT > 50% in 8 subjects who discontinued
			24 mo: 69.2 ± 43.5 [2.4 ± 1.5]	24 mo: 733 ± 497 [25.2 ± 17.1]	24 months: 10.6		Although both increased, neither PSA nor central prostate volume growth increased significantly; no change in IPSS score
Daily application of DHT gel (10 g of 0.7% DHT gel)	1	12, DHT gel	210 ± 20 [7.3 ± 0.7]	210 ± 50 [7.2 ± 1.7]	1	LC-MS/MS	No effects on serum lipids
							HDL did not change
Healthy men; age 35–55 (55)							All subjects had PSA <1.5-fold baseline at end of study, and none had a PSA >4.0 ng/mL at any time during the study
							Prostate volume and IPSS unaffected by DHT treatment

Abbreviations: DEXA, dual-energy X-ray absorptiometry; HCT, hematocrit; HGB, hemoglobin; IMT, initma-media thickness; sICAM, soluble intercellular adhesion molecule; sVCAM, soluble vascular cell adhesion molecule.

^*a*^ 18 enrolled.

^*b*^ 60 enrolled.

^*c*^ Calculated from T and DHT provided by authors.

### Transdermal DHT gel in middle-aged eugonadal men treated for 24 months

**Figure 2.**
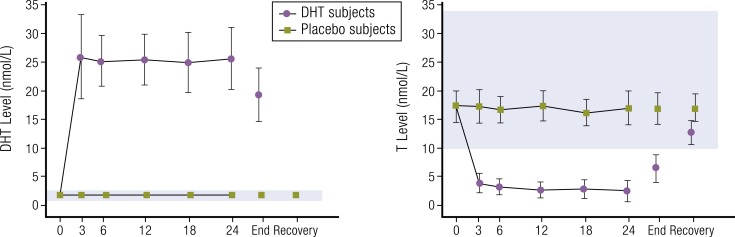
Mean (± standard error of the mean) serum DHT and T response to transdermal DHT therapy over 24 months of treatment in middle-aged men. Shaded region of each graph represents normal ranges for DHT or T. To convert T and DHT to ng/dL, values must be divided by 0.0347 or 0.0345, respectively. Redrawn from Idan *et al.* (54).

A placebo-controlled trial of DHT gel to evaluate the effect of DHT specifically on prostate growth rate has been published and is arguably the most significant report concerning the longer-term effects of supraphysiologic DHT exposure (54). DHT administration yielded a sustained increase in mean serum levels of DHT with a parallel decrease in mean concentrations of serum T. No changes in androgen levels were observed after placebo (Fig. 2). For men using DHT gel, mean serum DHT increased about 10-fold and mean serum T levels decreased by about 86% after 24 months of daily DHT gel application (Table 1).

The effect of sustained serum DHT levels resulted in only minor changes to serum PSA and prostate volume, none of which were statistically or clinically significant. Three men in the DHT-treated group were discontinued due to a rise in PSA to >4 ng/mL, but none was diagnosed with prostate cancer. One man in the placebo group required a transurethral resection of the prostate for BPH. Discontinuation of men treated with DHT occurred primarily due increased hematocrit (>50%), which was asymptomatic and resolved after stopping treatment. No serious adverse effects due to DHT occurred.

Overall, these studies of men treated with supraphysiologic doses of DHT do not support the hypothesis that modest elevations of DHT and DHT/T ratios observed with commonly used TRT preparations (including injectable T esters, transdermal T, and oral TU) will yield deleterious effects in men, particularly in androgen-sensitive tissues like prostate. Consistent with this conclusion are recent data from a longitudinal, observational cohort study of 3638 men in which circulating DHT (measured by LC-MS/MS) was not associated with incident prostate cancer (56).

## Association of Circulating Levels of DHT and DHT/T Ratio With Prostate Disease

Although androgens support the growth, proliferation, and progression of aggressive prostate cancer, there is no consensus that elevated levels of circulating androgens contribute to the risk of developing prostate cancer. On the contrary, there is strong evidence that circulating levels of DHT are not associated with increased risk of prostate cancer (57–59). This is because intraprostatic levels of androgens appear to be controlled by an internally regulated system that senses and adapts to circulating levels of T and DHT. So although it is possible (but not proven) that intraprostatic levels of T and DHT (along with estradiol) may play an important role in the development of prostate pathology, cross-sectional and longitudinal data do not demonstrate that elevated levels of circulating DHT increase the risk of prostate disease, even when high DHT levels or DHT/T ratios were sustained for long periods (54, 60).

It is generally accepted that intraprostatic DHT is derived primarily from the conversion of T to DHT by the enzyme SRD5A (61). Intraprostatic SRD5A activity is regulated by the SRD5A2 gene, and polymorphisms of this gene (particularly SRD5A2 V89L and A49T) have been studied for associations with prostate cancer risk. Notably, a recent meta-analysis of SRD5A2 gene polymorphisms and prostate cancer risk found that prostate cancer risk was not associated with V89L and was probably not associated with A49T (62). Furthermore, polymorphisms in CYP17 (MspA1) and SRD5A2 (V89L) genes have not been shown to increase serum T or androstanediol-glucuronide, a surrogate for upstream metabolism of the DHT (63). Thus, polymorphisms in genes associated with the synthesis of DHT do not appear to alter circulating levels. However, this has not been confirmed by the direct measurement of serum DHT in men with these polymorphisms.

In eugonadal men, the serum concentration of T probably plays a minor contributory role as a source for intraprostatic T. But in hypogonadal men, intraprostatic T concentrations are dissociated from circulating T concentrations (see Table 2). Marks *et al.* (66) reported that when hypogonadal men were treated with intramuscular T replacement for 6 months, average serum concentrations of T rose to about 640 ng/dL (22.19 nmol/L), whereas there was no significant effect on the intraprostatic levels of either T or DHT compared with baseline. There also was no effect of T therapy on prostate tissue biomarkers (*e.g.*, AR, Ki-67, or CD34) or gene expression (*e.g.*, AR, PSA, PAPA2, VEGF, NXK3, or clusterin). Lastly, there was no change in prostate histology or the incidence of prostate cancer or severity thereof, although this study was not powered for prostate cancer end points. Thus, at least for serum T, increased circulating levels have essentially no impact on intraprostatic androgen levels.

**Table 2.** **Serum and Intraprostatic DHT and DHT/T Ratios and PSA Observed in Response to Various Hormonal Manipulations**

Study Description	Length of Exposure	N	End-of-Rx Average Serum T (ng/dL) [nmol/L]	End-of-Rx Average Serum DHT (ng/dL) [nmol/L]	Mean Serum DHT/T Ratio	DHT Assay Method	Intraprostatic T (ng/g)	Intraprostatic DHT (ng/g)	Intraprostatic DHT/T Ratio	PSA Baseline/ End of Rx (ng/mL)
Case report: Pr CA in hypogonadism (64)	2 y TRT	1	19 [0.66]	2 [0.07]	0.10	LC-MS/MS	0.5	2.77	5.54	Not reported/49.0
Medical castration healthy men (65)	4 wk	4	357 ± 86 (SEM) [12.4 ± 3.0]	40 ± 14 [1.4 ± 0.5]	0.1	RIA	1.9 ± 0.3	9.1 ± 4	5	0.5 ± 0.2/0.5 ± 0.2
Double placebo										
Acyline plus placebo	4 wk	4	26 ± 14 (SEM) [0.9 ± 0.3]	8.72 ± 2.88 [0.3 ± 0.1]	0.33	RIA	0.4 ± 0.1	2.0 ± 0.5	3.5	0.8 ± 0.1/0.3 ± 0.1
Acyline plus T gel	4 wk	4	481 ± 167 (SEM) [16.7 ± 5.8]	140 ± 67 [4.8 ± 2.3]	0.30	RIA	1.5 ± 0.2	6.4 ± 0.8	4.5	0.8 ± 0.1/0.8 ± 0.1
DHT Rx (55)	4 wk	15	410 ± 50 (SD) [14.2 ± 1.7]	30 [1.03]	0.07	LC-MS/MS	0.6 ± 0.2	2.8 ± 0.2	4.6	1.1 ± 0.6/1.0 ± 0.5
Placebo										
DHT	4 wk	12	210 ± 20 (SD) [7.3 ± 0.7]	210 ± 50 [7.2 ± 1.7]	1	LC-MS/MS	0.4 ± 0.1	3.1 ± 0.5	7.75	0.7 ± 0.4/0.8 ± 0.5
TRT mild hypogonadism (66)	6 mo	19	273 (89–715)	26 (7–40)	0.09	RIA	0.88 (0.02–20.12)	5.10 (0.7–22.37)	5.6	0.97 (0–2.47)/1.10 (0.02–6.9)
Placebo										
TE	6 mo	21	640 (272–1190) [22.19]	47 (20–121) [1.62]	0.07	RIA	1.55 (0.1–23.1)	6.82 (1.57–39.82)	4.4	1.55 (0.3–5.8)/2.29 (0.4–7.1)
Male contraceptive trial (67)	10 wk	8	400 (median) [13.87]	50 [1.72]	0.12	LC-MS/MS	0.4 ± 0.6 (SD)	6.3 ± 1.9 (SD)	15.75	0.7 (0.6–1.0)/0.8 (0.7–1.12)
Placebo										
T gel	10 wk	7	440 (median) [15.26]	180 [6.20]	0.4	LC-MS/MS	0.7 ± 0.6 (SD)	6.0 ± 2.8 (SD)	8.57	0.7 (0.4–1.1)/0.9 (0.3–1.2)
T gel plus duasteride	10 wk	7	700 (median) [24.27]	50 [1.72]	0.07	LC-MS/MS	4.5 ± 1.5 (SD)	0.7 ± 0.2 (SD)	0.15	0.9 (0.7–1.1)/0.7 (0.7–1.1)

DHT measured by immunoassays without preparatory chromatography generally overestimate DHT levels and correlates poorly with LC-MS/MS data.

Abbreviations: Pr CA, prostate cancer; Rx, treatment; SD, standard deviation; SEM, standard error of the mean.

DHT and DHT/T ratios have been measured (or can be calculated) in a number of TRT clinical trials. The effects of various TRTs on prostate are summarized in Table 3. Although TRT has been associated with adverse prostate events, this table indicates that even striking elevations in DHT and DHT/T ratio for prolonged periods (*e.g.*, up to 24 months) have not been associated with clinically meaningful negative effects on prostate. However, it is important to emphasize that these trials were not designed and powered to detect long-term effects of elevated DHT on prostate tissue.

**Table 3.** **Serum DHT Concentrations and DHT/T Ratios Observed With Androgen Replacement Therapies and Reported Effects on Prostate**

Form of ART	Length of Exposure	N	Age	End-of-Treatment Mean Serum T (ng/dL) [nmol/L]	End-of-Treatment Mean Serum DHT (ng/dL) [nmol/L]	End-of-Treatment Mean DHT/T Ratio	DHT Assay Method	Observed Effects on Prostate	PSA or Change in PSA in Response to ART
Oral TU (CLR-610) (68)	28 d	15	46.7 ± 11	516 ± 58 [17.9 ± 2]	110 ± 15 [3.8 ± 0.5]	0.21	LC-MS/MS	None	Not reported
Nasal T gel (69)	90 d with 180- and 360-d extensions	BID: 228 TID: 78	54.4 ± 11	375-421 [13–15]	33–40 [1.14–1.38]	<0.1	LC-MS/MS	AE of PSA increased in six subjects in TID group at day 90	BID dosing:
									180 d: +0.01 ng/mL
									360 d: +0.06 ng/mL
									TID dosing:
									180 d: +0.09 ng/mL
									360 d: +0.21 ng/mL
Transdermal T gel (70, 71)	3 y	123	51.5 ± 0.9	432–577 [15–20]	130–210 [4.48–7.23]	0.26–0.30	RIA	AE of PSA increased in seven subjects; three with diagnosis of prostate cancer	Baseline:
									0.85 ± 0.06 ng/mL
									6 mo:
									1.11 ± 0.08 ng/mL (with no further significant increase)
Transdermal T solution (72)	120 d	155	51.5	389–507 [13.5–17.6]	98 [3.37]	0.17–0.26	LC-MS/MS	AE of PSA increased in one subject with diagnosis of prostate cancer	Mean increase of 0.02 μg/L
Scrotal T patch (73)	8 y	25	Not reported	404 [14]	175 [6.03]	0.43	RIA	None	Not reported
Nonscrotal	24 wk	33	44.3 ± 11.1	564 ± 149 [19.6 ± 5.2]	50 ± 20 [1.72 ± 0.7]	0.09	RIA	AE of one subject with diagnosis of prostate cancer	Wk 24: no change from baseline
T patch									
Parenteral TE (74)		33	44.9 ± 11.6	812 ± 181 [28.2 ± 6.3]	66 ± 26 [2.3 ± 0.9]	0.08		AE of one subject with diagnosis of prostate cancer	Baseline:
									0.9 ± 0.7 ng/mL
									Wk 24:
									1.4 ± 2.2 ng/mL
Oral TU, 80 mg BID	Several months	5	Range, 60–72	233 ± 148 [8.06 ± 5.13]	93 ± 42 [3.20 ± 1.46]	0.40	RIA	None	Not reported
DHT gel, 125 mg BID (46)		12		98.1 ± 94 [3.4 ± 3.26]	520 ± 272 [17.9 ± 9.38]	5.3			
Oral TU, 80–200 mg/d (75)	10 y	33	Range, 15–62	188 ± 40.4 [6.5 ± 1.4]	90 ± 41 [3.1 ± 1.4]	0.48	RIA	None	Measured during last 2 y only; within normal limits
T pellets (1200 mg in single s.c. dose)*^a^* (76)	300 d	14	32.77 ± 2.59	742 ± 48 [25.7 ± 1.7 ]	145 ± 18 [4.9 ± 0.62]	0.20	RIA	Not reported	Not reported
Transdermal DHT gel (70 mg DHT/d) (51)	3 mo	17	68.2 ± 1.15	144 ± 57 [4.99 ± 1.98]	534 ± 99 [18.4 ± 3.4]	3.7	RIA	None	Mean increase of 1.0 ng/mL
Transdermal DHT gel (125–250 mg DHT/d) (52)	6 mo	54	58.4 ± 5.3	170 ± 1112 [5.89 ± 3.88]	238 ± 133 [8.19 ± 4.58]	1.4	RIA	None	No change
Transdermal DHT gel (70 mg/d) (54)	24 mo	56	60.5 ± 0.7	69.2 ± 43.5 [2.4 ± 1.5]	733 ± 497 [25.2 ± 17.1]	10.6	LC-MS/MS	None	Mean increase of 0.2 ng/mL
Parenteral TE	5 mo	11	Young: 18–35	550 [19.01]	125 mg TE/d: 50 ± 2.5 [1.72 ± 0.09]	0.09	LC-MS/MSRIA*^c^*	Not reported	Not reported
Weekly dose of 125 mg (hypogonadism induced with GnRH agonist)*^b^* (9)		11	Older: 60–75	778 [26.9]	125 mg TE/d: 70 ± 5.0 [2.41 ± 0.17]	1.0		Not Reported	Not Reported
Parenteral TU (750 mg TU at 0 and 4 weeks and then every 10 weeks) (77)	84 wk	93	54 ± 0.9	495 ± 142 [17.2 ± 4.9] (*C*_avg_ days 0–70 after third injection)	25 ± 10 [0.86 ± 0.34]	0.05	LC-MS/MS	AE of one subjects with diagnosis of prostate cancer	Baseline: 1.0 ng/mL
									84 weeks: 1.4 ng/mL

Abbreviations: AE, adverse event; ART, androgen replacement therapy; BID, twice daily; C_avg_, average T concentration; s.c., subcutaneous; TID, three times daily.

^*a*^ Calculated during period when serum T in response to T pellets was measured and in the eugonadal range (*i.e.*, between 21 and 175 days after dosing).

^*b*^ TE dosages of 25, 50, 125, 300, and 600 mg/wk evaluated, but only data for 125 mg/wk included in this table as this is the typical dosage used for the treatment of adult male hypogonadism.

^*c*^ Correlation between LC-MS/MS and RIA assay was 0.99.

In addition, we have been unable to identify a single epidemiological study that has implicated serum DHT as a factor positively associated with an increased risk of prostate cancer. Data from 18 prospective studies that included 3886 men with incident prostate cancer and 6438 control subjects were pooled and analyzed by the Endogenous Hormones and Prostate Cancer Collaborative Group (78) in an effort to determine what associations, if any, existed between serum androgens (among other factors) and prostate cancer. Results from this analysis failed to identify any correlation with DHT (nor with the terminal metabolite of DHT, androstanediol-glucuronide) and prostate cancer. Given the potential for the prostate gland to regulate intraprostatic concentrations of T, DHT, and estradiol, along with metabolism of these hormones, this finding is not surprising. Moreover, it is becoming increasingly clear that intraprostatic genetic control mechanisms and genetic susceptibility to gene mutations, translocations, and various loci recently identified (79–82) are responsible for such control. These genetic events are beyond the scope of this review but are likely to be much more important in prostate cancer risk than circulating levels of T or DHT.

Based on our review of the available DHT safety data in young and older men (the majority of which is included in this review), we conclude that the modest increase in DHT concentrations and DHT/T ratios commonly associated with TRT pose a low probability of risk for prostate disease. And although long-term safety evaluations appropriately powered to assess disease end points (including prostate cancer and urinary retention) are needed to formally evaluate this risk, such studies will be problematic given the challenge of evaluating DHT effects in the presence of other endogenous androgens, most notably T. To this end, use of an injectable or transdermal DHT preparation in a prospectively designed outcomes study merits consideration.

## Intraprostatic Control of DHT in the Presence of Fluctuating Levels of Circulating Androgens

The prostate is not a passive recipient of circulating T and DHT but rather has the ability to synthesize and metabolize these androgens. Therefore, except when serum T levels are extremely low, intraprostatic DHT levels are primarily controlled by intraprostatic factors rather than circulating T and DHT levels. To understand the various paths by which DHT can accumulate in the prostate, we briefly review here T and DHT synthesis and metabolism, and the evidence that these intraprostatic pathways are the primary controls of intraprostatic DHT levels.

### DHT sources in the prostate

**Figure 3.**
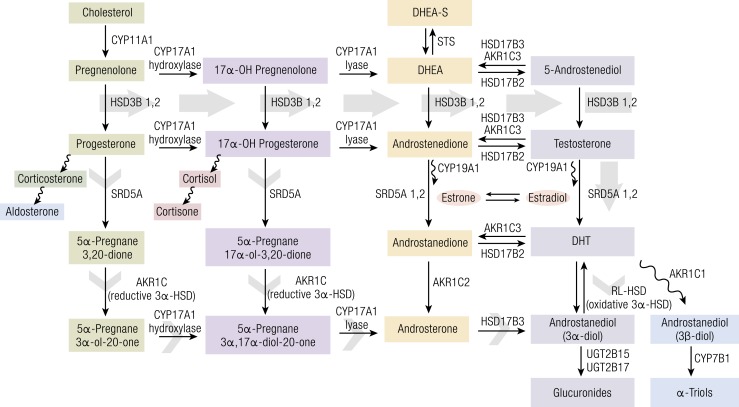
The classical and “back-door” pathways of androgen biosynthesis. In the classical pathway (solid gray arrow), C21 precursors (pregnenolone and progesterone) are converted to the C19 adrenal androgens dehydroepiandrosterone (DHEA) and androstenedione by the sequential hydroxylase and lyase activities of CYP17A1. Circulating adrenal androgens [including dehydroepiandrosterone-sulfate (DHEA-S)] enter the prostate and can be converted to T by a series of reactions involving the activity of HSD3B, HSD17B, and aldo/keto reductase (AKR1C) enzymes. T is then converted to the potent androgen DHT by the activity of SRD5A. In the back-door pathway to DHT synthesis (short gray arrows), C21 precursors are first acted upon by SRD5A and the reductive 3*α*-hydroxysteroid dehydrogenase (3*α*-HSD) activity of AKR1C family members, followed by conversion to C19 androgens via the lyase activity of steroid 17*α*-monooxygenase (CYP17A) and subsequently to DHT by the action of HSD17B3 and an oxidative 3*α*-HSD enzyme. Redrawn from Mostaghel and Nelson (4).

Although DHT enters many tissues through diffusion from the systemic circulation, DHT in the circulation does not diffuse into the prostate because DHT concentrations in the prostate are markedly higher than the systemic circulation (intraprostatic DHT is on average 6- to 10-fold higher than circulating DHT) (83, 84). The vast majority of DHT in the prostate is derived from three sources: (1) the classical pathway whereby testicular and adrenal T diffuses into the prostate and is converted, *in situ*, into DHT by SRD5A (shown in solid gray arrow in Fig. 3); (2) synthesis directly from 17-hydroxypregnenolone and 17-hydoxyprogesterone (known as the backdoor pathway and shown in short gray arrows in Fig. 3); and (3) intracrine reverse synthesis (back conversion) from the DHT metabolite 5*α*-androstane-3*α*,17*β*-diol (3*α*-diol) through the oxidative function of 3*α*-HSD (upward arrow in Fig. 3). The prostate also can metabolize DHT to inactive glucuronides by various irreversible pathways (see Figs. 1 and 3). The control of these processes undoubtedly plays a role in regulating DHT levels in prostate tissue and, more specifically, in certain cell types within prostate. In addition, some DHT may enter the prostate if it is bound to SHBG because megalin on prostatic cells can bind SHBG and transport the DHT-SHBG complex into the cell (28). However, the contribution of this pathway is considered to be relatively modest (85). Collectively these various pathways of DHT synthesis and metabolism, many of which are tightly regulated, maintain a steady DHT/T ratio in the prostate cells that is relatively indifferent to high or low circulating DHT levels.

### Impact of changes in systemic T and DHT on intraprostatic T and DHT in hypogonadal men

Prostate cancer occurs in men with low circulating T, and it has been estimated that 14% to 35% (86, 87) of men with prostate cancer are hypogonadal at the time of diagnosis. Furthermore, and for reasons that are not understood, it has been reported (reviewed in Raynaud) (88) that low T levels are associated with higher Gleason scores on prostate core biopsies and positive surgical margins after prostatectomy (86–89). These data suggest that low circulating T and DHT levels do not lower the risk of prostate cancer and, in fact, may predispose to more aggressive tumors, supportive of the concept that intraprostatic synthesis of DHT can come from sources other than circulating T. An alternate explanation is that SRD5A is so finely modulated that intraprostatic DHT levels only fall when the substrate (*i.e.*, T) is very low. This possibility is discussed later. From an oncology perspective, regardless of the mechanism(s) at play in prostate that control DHT synthesis, the fact that DHT can be synthesized within prostate tissue helps to explains why androgen deprivation therapy (ADT) is not totally effective in controlling prostate cancer. Notably, blockade of residual androgen synthesis (including DHT) through all pathways mentioned previously by abiraterone (CYP17 inhibitor) has been shown to prolong survival in men with prostate cancer (90, 91).

### Hypogonadism and intraprostatic DHT and T

A case report of prostate cancer that developed in a 74-year-old man who underwent bilateral orchiectomy at age 5 for testicular trauma illustrates the point that intraprostatic DHT is independent of circulating levels (64). In this patient, the circulating levels of T and DHT were low [19 and 2 ng/dL (0.66 and 0.07 nmol/L), respectively], whereas intraprostatic levels of androstenedione, T, and DHT were similar to the T and DHT levels in the prostate of eugonadal men who had prostatectomies for prostate cancer. Moreover, his intraprostatic DHT level was comparable to the intraprostatic DHT levels of normal individuals in the placebo arm of the DHT gel study (Table 2). These findings demonstrate that circulating levels of DHT (in this case at the very low end) are not reflected by intraprostatic levels. The mechanism for this likely involves the expression in prostate tissue of STS (steroid sulfatase), HSD3B2 (hydroxysteroid dehydrogenase), AKR1C3 (prostate-specific 3-, 17-, and 20-ketosteroid reductases), and SRD5A1 and SRD5A2 [5-AR(5*α*-reductase) types I and II] (see Fig. 3) that enables prostate tissue to synthesize DHT from intraprostatic dihydroepiandrosterone (DHEA) rather than from adrenal androgens by the classical pathway.

### Induced hypogonadism in healthy volunteers and the effect on intraprostatic T and DHT levels

The effect of androgen deprivation and replacement on intraprostatic androgen levels has been evaluated in healthy volunteers (65). Subjects (N = 16) were randomized to one of three treatment arms for 28 days: control [placebo GnRH antagonist (acyline) injection, placebo gel daily], ADT therapy [GnRH (gonadotropin-releasing hormone) antagonist injection, placebo gel], and ADT therapy and T replacement (GnRH antagonist injection, 100 mg transdermal T gel daily).

In all three groups, the intraprostatic DHT/T ratios were similar (3.5 to 5), with the intraprostatic DHT level varying about 4.5-fold (Table 2). This variation in intraprostatic DHT does not parallel the almost 16-fold swing in circulating DHT nor the almost 19-fold negative swing in serum T concentrations. In men treated with ADT and placebo gel, there was an approximate 78% decrease in intraprostatic levels of T and DHT compared with placebo. This effect occurred due to a decrease in circulating T because the magnitude of the decrease in men treated with ADT and T was substantially less (approximately 25%). In spite of this, there was no correlation in the ADT-treated group between serum T and DHT levels and corresponding intraprostatic androgen levels. The intraprostatic DHT in the medically castrate group remained 20-fold higher than values observed in serum and comparable to levels of serum T in placebo-treated men. According to the authors, the absolute values of intraprostatic androgens in this study are somewhat higher than values reported in men with BPH and cancerous prostate tissue.

### Short-term DHT treatment and intraprostatic T and DHT levels in healthy volunteers

In a double-blind, randomized, placebo-controlled study, exogenous DHT had little impact on intraprostatic androgen levels (55). A prostate biopsy was performed 28 days after daily treatment with a transdermal DHT gel (70 mg DHT) in healthy men. DHT administration led to a robust sevenfold increase in mean serum DHT levels rising from 26 to 210 ng/dL (0.90 to 7.23 nmol/L) at day 28 (Table 2). T levels decreased significantly and the serum DHT/T ratio increased to 1.0. In spite of the huge increase in serum DHT concentration, intraprostatic androgens were unaffected. Furthermore, intraprostatic levels of T and DHT in DHT-treated men did not differ significantly from placebo. Monitoring of PSA did not show differences during the course of treatment, and prostate volume assessed by transrectal ultrasound also did not change. Finally, gene expression analysis of RNA extracted from the prostate biopsies did not show differences between the placebo arm and the DHT gel arm, even in androgen-responsive gene messages (55). These findings are consistent with those from a study in hypogonadal men treated with T (67) where prostate-specific microarray analysis performed on tissues with the highest T and DHT levels [and confirmed by reverse transcription polymerase chain reaction (RT-PCR)] did not reveal any significant changes in 234 genes known to be androgen regulated.

### Male contraceptive trial with T gel and T gel plus dutasteride in healthy males: intraprostatic androgen levels

A single-blind, randomized, placebo-controlled trial was conducted in a single center to determine the impact of male hormonal contraception on intraprostatic androgen levels after 12 weeks of treatment. Eligible subjects were randomized to placebo, transdermal T gel, T gel plus depot medroxyprogesterone acetate, or T gel plus dutasteride (a potent inhibitor of SRD5A) (67). Primary end points included intraprostatic androgen levels and indices of androgen effects on the prostate, including biomarkers and microarray analysis.

The substantially elevated median serum DHT level of 180 ng/dL (6.20 nmol/L) in the T gel arm and a DHT/T ratio of 0.4 did not impact intraprostatic T or DHT levels (Table 2). Notably, the addition of the 5AR-I dutasteride led to a significant increase in both serum and intraprostatic T (11-fold) and a decrease (–90%) in intraprostatic DHT compared with placebo. There were no statistical differences in PSA or prostate volumes between these three arms. The microarray data of androgen-regulated genes, along with confirmation by RT-PCR, showed similar levels of expression and no statistically significant differences between placebo and all other treatment arms.

### Brief review of intraprostatic T and DHT levels in ADT-treated prostate cancer patients

In two reviews addressing intraprostatic T and DHT levels in either BPH or prostate cancer, there were wide variations in intraprostatic DHT levels but no demonstrable differences in intraprostatic DHT levels between normal prostatic tissue, BPH tissue, and prostate cancer tissue. More relevant to this discussion is a study that explored intraprostatic androgen response in patients with BPH treated with 5AR-Is and patients with prostate cancer treated with ADT (92, 93). Despite striking reductions in serum DHT and/or T levels, the intraprostatic androgen levels were above the threshold required for activation of AR.

**Figure 4.**
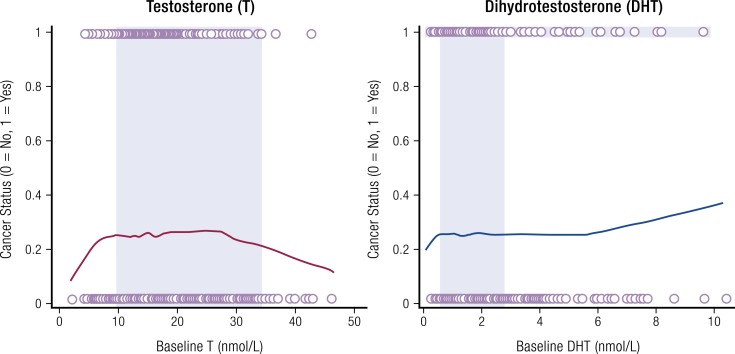
Results from REDUCE trial showing cancer risk vs baseline serum androgen concentration. Locally weighted scatterplot smoothing of serum levels of T and DHT at baseline and final cancer status after considering all biopsies during 4 years of the REDUCE trial. The overlapping circles on the top and bottom of the chart represent each individual case. Cancer risk with individuals overlapping circles of subjects with cancer were scored as 1, whereas subjects without cancer were scored as 0. Shaded regions of each graph depict eugonadal ranges for T and DHT. To convert T and DHT to ng/dL, divide by 0.0347 or 0.0345, respectively. Redrawn from Muller *et al.* (3).

Additional evidence in favor of intraprostatic androgen control was observed in the REDUCE trial (Reduction by Dutasteride of Prostate Cancer Events) (3). REDUCE was a large, prospective, 4-year, double-blind, placebo-controlled, randomized clinical trial of a 5AR-I (dutasteride) for risk reduction of biopsy-detectable prostate cancer. Among men with prostate cancer, T and DHT levels were not statistically different regardless of Gleason score. Furthermore, no association was found between quintiles of either androgen with risk of low- or high-grade cancer except for the second quintile of DHT, which was associated with a lower risk of low-grade prostate cancer. Both T and DHT were also disassociated from low- and high-grade prostate cancer when tested continuously or as a trend across all concentration quintiles. As shown in Fig. 4, it is noteworthy that cancer status (*i.e.*, risk) did not change appreciably over wide serum T and DHT concentrations [*i.e.*, from approximately 250 to 900 ng/dL (8.67 to 31.20 nmol/L) for T and from 15 to 175 ng/dL (0.52 to 6.03 nmol/L) for DHT]. This can be visually appreciated by the shaded areas on Fig. 4 that show a close correlation between common reference ranges for T and DHT and the plateau for cancer risk for each androgen. Even at DHT concentrations >175 ng/dL (>6.03 nmol/L), the increased risk of prostate cancer was small. Finally, it should be mentioned that data from the placebo arm of the Prostate Cancer Prevention Trial in which serum DHT was measured over the course study also revealed no association between serum DHT concentrations and prostate cancer (3). DHT was not measured in subjects treated with finasteride.

Improved survival in men with castration-resistant prostate cancer who were treated with abiraterone (a CYP17A inhibitor) (91) also supports the concept that androgen synthesis within the prostate can totally bypass DHT synthesis from T in peripheral tissues. In this case, DHT is synthesized predominantly from adrenal precursors and intraprostatic DHT synthesis through the backdoor pathway (Fig. 3). When the backdoor pathway was suppressed by a steroidogenic enzyme blocker, namely, abiraterone, prolonged survival was observed. Collectively, these data support the notion that circulating T and DHT are likely of little relevance with respect to development of prostate cancer compared with intraprostatic levels of these two hormones.

## Do Increases in Circulating Levels of DHT Increase Risk of CVD?

### Clinical data from DHT administration in supraphysiologic doses on CVD

Aside from TRT preparations, which modestly raise serum DHT concentrations and DHT/T ratios (described in Section X), there are three double-blind, placebo-controlled trials (see “Serum DHT and DHT/T Ratios Observed in Response to Testosterone Therapy in Men With Low T”) in which men have been treated with transdermal DHT gel. In all of these studies, DHT treatment resulted in sustained increase serum DHT to high supraphysiologic levels of DHT [*e.g.*, in the range of 700 ng/dL (24.27 nmol/L)] for up to 24 months (Table 1). Although these trials were small and not powered for detecting CV safety signals, there were no serious cardiovascular events reported in men who were exposed to exceptionally high serum DHT and DHT/T ratio. In the Idan *et al.* study (54), where eugonadal men were treated with DHT for up to 24 months, DHT therapy was not associated with a change in right carotid intima-media thickening, a sensitive predictor of future cardiovascular disease (CVD) and stroke risk (94). The only significant adverse events that were CVD related in the DHT group were pericarditis and atrial fibrillation (one subject) and single occurrences of pulmonary embolism and deep vein thrombosis. These were not deemed treatment related by the investigators. DHT exposure did not alter serum cholesterol, including circulating low-density lipoprotein (LDL) or high-density lipoprotein (HDL).

### Epidemiologic data exploring association of DHT with CVD risk

**Figure 5.**
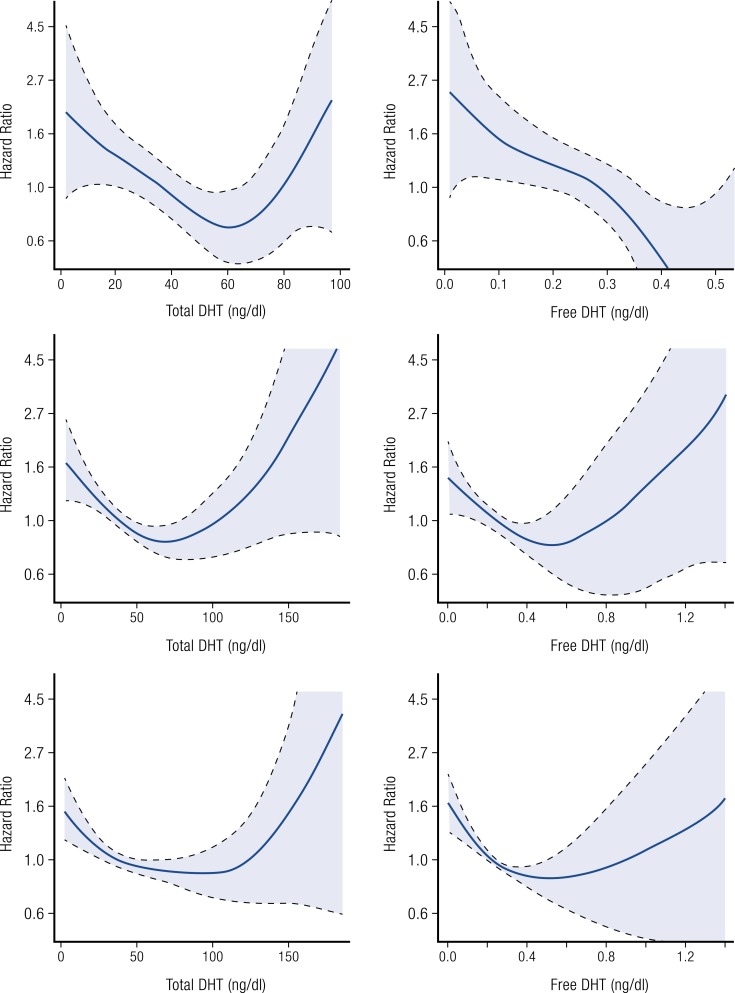
Regression graphs depicting the associations between levels of total and free DHT and ischemic stroke risk (Panel A), incident CVD risk (Panel B) and all-cause mortality risk (Panel C) in older normal men. Redrawn from Shores *et al.* (95, 96).

A longitudinal cohort study evaluated whether total T, calculated free T, DHT, and calculated free DHT were associated with incident CVD and mortality in eugonadal men in the Cardiovascular Health Study (mean age, 76 years; range, 66 to 97 years) who were free of CVD at the time of blood collection (95). Hormone concentrations were measured by LC-MS/MS. The authors concluded that DHT and calculated free DHT were associated with incident CVD and all-cause mortality. However, most events clustered into the midnormal DHT range with few events at low or high DHT levels, thus necessitating the use of a curvilinear model that resulted in wide confidence intervals (CIs) (Fig. 5). The authors noted that a causal relationship between DHT and CVD could not be determined and that prospective studies are needed to confirm these results and to clarify the underlying physiologic mechanisms.

In a further analysis from the Cardiovascular Health Study, the relation of DHT and stroke and mortality was assessed (96). Figure 5 shows that total DHT had a nonlinear association with stroke and incident CVD [*i.e.*, myocardial infarction, stroke, or CVD death) and all-cause mortality, where low (<50 ng/dL (<1.72 nmol/L)] and high [>74 ng/dL (>2.55 nmol/L)] levels of DHT were shown to be associated with risk of incident CVD whereas midrange DHT values [50 to 74 ng/dL (1.72 to 2.55 nmol/L)] were not. An intriguing finding in this study was that high free DHT levels nullified this relationship as free DHT was found to be negatively associated with CVD risk in a linear fashion. Because most events clustered into the midnormal DHT range with few events at low or high DHT levels, use of a curvilinear model was required that resulted in wide CIs at low and high DHT concentrations. Total T and free T showed no association with stroke risk. There is no obvious biological explanation for the discrepancy between the free DHT and total DHT relationships with stroke. The epidemiologic data are inconclusive as to a causal relationship of DHT with CVD end points. These relationships need to be further explored in other epidemiologic cohorts and prospective randomized trials of sufficient scale to be meaningful.

Results of a meta-analysis to explore whether the incidence of CV events is affected by the mode of TRT administration have been recently published by Borst *et al.* (97). A secondary focus of this study was to determine if there was a differential elevation of serum T vs DHT and whether CV risk was impacted by changes to either androgen. Among any men who received any form of TRT, the estimated risk ratio for CV events was 1.28 (95% CI: 0.76 to 2.13). Compared with placebo, this was not statistically significant (*P* = 0.34). In contrast, when CV rates were analyzed on the basis of route of administration, oral T (as a class) resulted in a significant increase in CV events (estimated risk ratio = 2.20; 95% CI: 1.45 to 3.35; *P* = 0.015 vs placebo). Unfortunately, the number of oral studies identified by the authors as meeting the *a priori* inclusion criteria was very small (*i.e.*, four), as was the number of subjects per study. In addition, three of the four oral studies evaluated androgen response in men with normal T levels and two of these studies were conducted in men with serious comorbid conditions, namely, malnourishment and alcoholic cirrhosis. Furthermore, and as noted by Borst *et al.*, the methodology used to assay serum DHT at the time the oral T studies were conducted used immunoassays that may have overestimated serum DHT levels. For these reasons, significant caution must be exercised when considering these data.

In contrast to the findings of Borst *et al*. (97), two separate studies, one a cross-sectional analysis (98) and the other a longitudinal analysis (99), have assessed associations of T, DHT, and estradiol with all-cause ischemic heart disease and lower-limb intermittent claudication in older (70 to 89 years) men. Higher DHT levels (measured by LC-MS/MS) were associated with lower risk for intermittent claudication and ischemic heart disease, independent of SHBG concentrations and conventional risk factors for vascular disease. The authors concluded that their findings were consistent with a possible cardioprotective influence of androgen exposure but that additional prospective studies are needed to confirm the observed effects.

Finally, differential effects of T, DHT, and estradiol on carotid intima-media thickness and the presence of carotid plaque in men with and without coronary artery disease (CAD) have been reported (100). In men with CAD, higher DHT or estradiol was associated with significantly less carotid plaque, but this was not the case in men without CAD. Here, T was associated with reduced carotid intima-media thickening and a lower prevalence of carotid plaque, whereas estradiol was associated with opposite effects. The potential role of DHT to influence risk of CAD, perhaps by directly affecting adverse pathological processes involved in the development of atherosclerosis, deserves significantly more investigation in well-controlled interventional clinical studies in men at risk for CAD but without frank clinical manifestations of CVD as well as men with CAD.

“In men with coronary artery disease, higher dihydrotestosterone or estradiol were associated with significantly less carotid plaque.”

### Effect of DHT treatment in men with CAD

The effect of transdermal DHT (32 mg DHT gel applied daily to the abdominal area) on left ventricle mass and its systolic and diastolic function as well as on the results of a treadmill stress test was assessed in 11 eugonadal men (mean age, 58.5 years) and in age-matched control subjects with a prior history of myocardial infarction (101, 102). Control subjects received placebo gel. Serum DHT data were not provided, but in an effort to provide a rough estimate of DHT response, we note that transdermal administration of this dose and DHT formulation to the same body location in older normal men by Wang *et al.* (103) yielded mean serum DHT levels at day 14 of 348 to 493 ng/dL (12 to 17 nmol/L) at steady state (about 3 days after once-daily administration). Compared with untreated controls subjects, 3 months of DHT treatment resulted in a significant decrease in isovolumetric relaxation time (0.150 ± 0.37 seconds vs 0.135 ± 0.03 seconds; *P* < 0.05) compared with controls, indicating the improvement of left ventricle diastolic function. Left ventricle mass and systolic function indices remained unchanged. There was improvement in myocardial ischemia, time to 1-mm ST segment depression increased (*P* < 0.05), and ST segment/heart rate slope decreased (*P* < 0.01). DHT treatment was also associated with a significant (60%; *P* < 0.01) decrease in chest pain during electrocardiogram stress testing. These changes reflect a significant improvement in coronary reserve in response to a direct vasodilatory effect of DHT and are consistent with similar findings for T (104). The DHT regimen did not alter LDL or HDL. In addition, no changes were observed in blood glucose, insulin, insulin resistance (homeostasis model assessment), and fibrinogen. A tendency toward higher levels of hemoglobin, erythrocyte count, and hematocrit [45.1 ± 6.0 (baseline) to 47.2 ± 6.0 (3 months)] did not reach statistical significance. Overall, DHT therapy in men with CAD decreased myocardial ischemia and improved left ventricular diastolic function. Because this study was small in scope, larger, placebo-controlled studies should be conducted to confirm these positive findings.

### Effect of oral TU and resultant elevated DHT in hypogonadal men with CAD

The effects of an oral TU formulation [80 mg TU, twice daily (BID)] on myocardial perfusion and vascular function in hypogonadal men (mean age, 57 years) was examined in a placebo-controlled, cross-over–designed study in a modest number of men (N = 25) with CAD (104). Oral TU significantly increased serum DHT from a baseline of 34 to 64 ng/dL (1.17 to 2.20 nmol/L) after 8 weeks. In response to oral TU, myocardial perfusion in myocardium perfused by unobstructed CAD increased, whereas perfusion in areas of myocardium supplied by coronary arteries with significant atheroma was not affected. TU treatment also decreased peripheral and central arterial stiffness and, concurrently, modestly increased left ventricular ejection fraction. Overall, the effects of oral TU and its associated increase in DHT were small, but it is noteworthy that in a relatively high-risk patient population, modestly elevated serum DHT was not associated with a worsening of CV status over a relatively short period of exposure. In light of the fact that this study was not powered for CV outcomes, these findings also merit confirmation in a larger, well-controlled trial.

“Alternate synthetic pathways may have particular clinical significance within prostate tissue.”

“Newer formulations of oral testosterone undecanoate for testosterone replacement therapy are in development.”

### Effects of DHT on various biomarkers of CVD risk

#### Vasodilatory effects of DHT in animal models and effects on endothelial nitric oxide synthase (eNOS) generation

Here we briefly review existing *in vivo* animal data or endothelial cell culture experiments that explore the role of androgens (most notably, DHT) on endothelial cell function. Goglia *et al.* (105) showed that physiologic doses of T and DHT given to normal or ovariectomized Wistar rats *in vivo* or in human aortic endothelial cell cultures *in vitro* increase the synthesis of nitric oxide through eNOS phosphorylation via the ERKPI3K/AKT pathway. Although DHT exerts these actions through the AR, T acts, in part, through aromatase-dependent conversion to estradiol. T and DHT also increased the tissue plasminogen activator/plasminogen activator inhibitor ratio favoring fibrinolysis. Yu *et al.* independently confirmed the mechanistic action of T and DHT on phosphorylation of eNOS through the PI3K/AKT pathway using the same cell culture system (106).

A study by Norata *et al.* (107) demonstrated that DHT inhibited the tumor necrosis factor-*α* and lipopolysaccharide-induced expression of vascular cell adhesion molecules (VCAMs) and intercellular adhesion molecules (ICAMs). In addition, DHT inhibited messenger RNA (mRNA) expression of IL-6, PAI-1, and Cox-2 and the release of cytokines and chemokines such as growth-regulated oncogene proteins (GRO), granulocyte-macrophage colony-stimulating factor, and tumor necrosis factor in endothelial cell culture. The DHT effect was counteracted by bicalutamide, an antagonist of the AR, thus confirming a direct effect of DHT. Androgen stimulation of nitric oxide production in human endothelial cells was also reported by Campelo *et al.* (108). These authors used T in conjunction with finasteride and an aromatase inhibitor and found that the T effect was partially mediated by DHT, whereas estradiol played no role in this process.

In summary these *in vitro* data show that the T and DHT (via their anti-inflammatory effects) preserve endothelial cell function and prevent synthesis of cell adhesion molecules and release of proinflammatory cytokines. These findings could explain some of the previously described clinical observations of the relationship between low T and DHT and peripheral vascular disease and the anti-ischemic effects of acute infusion of T in men with CAD and similar effects by DHT gel treatment (101, 104).

#### Evidence of DHT-mediated inhibition of macrophage foam cell formation

DHT has been shown to prevent macrophage foam cell formation in preclinical models. Ahmadi *et al.* demonstrated the presence of high-affinity ARs in a variety of types of macrophages and showed that DHT in pharmacologic concentrations inhibits formation of IL-6 (109). However, the most intriguing observations are the *in vivo* effect of DHT on foam cell formation in New Zealand rabbits fed a high-cholesterol diet (HCD). In this experiment (110), rabbits were divided into four groups: (1) sham operated but noncastrated fed regular chow diet; (2) castrated and fed normal chow diet; (3) castrated and fed HCD diet plus placebo implant; and (4) castrated with DHT implant and fed HCD diet. Plaque area was assessed in the entire aorta after 8 weeks. Microscopic examination of the aorta revealed that compared with the placebo group (group 3), DHT significantly reduced HCD-induced foam cell formation. This effect was accompanied by marked inhibition of LOX-1 mRNA (one of the ox-LDL receptors). In other *in vitro* experiments, ox-LDL (a potent atherogenic lipid) failed to induce foam cell formations from macrophages in the presence of DHT. If the macrophages were from AR knockout mice, DHT did not block foam cell formation. Thus, at least in this animal model, DHT inhibited ox-LDL–induced foam cell formation and atherosclerosis.

#### Effects of DHT therapy on human inflammatory biomarkers

Ng *et al.* (53) evaluated the effect of DHT gel (70 mg daily) vs placebo therapy on serum inflammatory markers in older men (>60 years) with partial androgen deficiency. At the 3-month time point, mean serum T had decreased from 432 ng/dL (14.98 nmol/L) to 230 ng/dL (7.97 nmol/L) and DHT increased from 42 ng/dL (1.45 nmol/L) to 733 ng/dL (25.24 nmol/L) in the DHT gel group. The DHT/T ratio was about 3.2-fold higher than the baseline value of 0.10. DHT therapy had no effect on levels of the inflammatory markers, namely, high sensitivity C-reactive protein (hs-CRP), ICAM-1, and VCAM-1. These data provide a measure of reassurance that increases in DHT and DHT/T ratio do not upregulate cellular mediators of inflammation or cell adhesion molecules.

#### Effects of DHT on EPCs

Endothelial progenitor cells (EPCs) are believed to play an important role in the maintenance and repair of injured endothelium and are negatively correlated with cardiovascular outcomes, including coronary heart disease (CHD)-associated mortality (111, 112). Although there is conflicting evidence regarding the role of androgens on EPCs in favor of an estrogen-mediated action, recent research demonstrates a positive action of androgens on EPCs. Of particular note is strong evidence that DHT dose-dependently augments the proliferation, migration, adhesion, and colony-forming activity of EPCs through AR-dependent (113, 114) and P13K/Akt, RhoA/ROCK, and possibly Erg 1 signaling pathways (114, 115). Additional angiogenesis genes upregulated by DHT include Vcan and Efnb2, whereas Cdk2ap1 is downregulated (thus promoting EPCs via cell cycle activation). Together, these data suggest that DHT may play an important role in endothelial health, a role that may help explain why free DHT (but not total DHT) has been negatively correlated with the incidence of CVD in men (see “ Epidemiologic Data Exploring Association of DHT With CVD Risk”). With respect to hypogonadal men, it is noteworthy that EPCs in this population are low and that TRT is associated with significant increases in EPCs that may be driven, at least in part, by the actions of DHT (116, 117). Further evaluation of DHT in blood vessel pathologies is merited, particularly in untreated hypogonadal men who, by nature of their T status, may be at risk for CVD (118).

#### Effects of DHT on platelet aggregation and thrombosis

There has been a long-standing concern regarding androgen use and its potential relationship to thrombosis, an area that remains controversial (119, 120). We will not review here the general literature in this regard but instead focus on the limited data regarding DHT based on animal and human clinical data. The processes involved in thrombosis are complex and reflect the integrated response of pro- and antithrombotic mediators as well as complex interactions of androgen and estrogen that are poorly understood. Evidence that DHT may act to stimulate platelet aggregation was first noted in mice implanted with DHT pellets. However, the effect of DHT was only observed in mesenteric arterioles and not, *ex vivo*, in platelet aggregation experiments (121). In rat studies, physiologic concentrations of DHT [2 nmol/L (58 ng/dL)] were shown to significantly inhibit adenosine 5′-diphosphate–induced platelet aggregation via direct interaction with ARs in platelets. At 291 ng/dL (10 nmol/L), this effect was lost but DHT did not stimulate aggregation to a greater extent than that observed in control or castrated rats (122). A similar action of DHT at 58 ng/dL (2 nmol/L) was observed in oxidative stress–induced platelet aggregation that was also associated with a reduction in thromboxane A2 release from platelets (123).

Clinical studies of DHT therapy in men have not revealed a demonstrable effect of sustained supraphysiological levels of DHT on thrombosis or endothelial function (See “ Clinical Data From DHT Administration in Supraphysiologic Doses on CVD” for detail). DHT therapy for 6 months did not adversely affect endothelial and smooth muscle–dependent vascular function as measured by flow-mediated or glycerol trinitrate–induced dilation nor was brachial artery size affected (51). In a 24-month trial of DHT therapy in normal men, there were no thrombotic events attributed to DHT nor were exceptionally high levels of DHT associated with a change in right carotid intima-media thickening (54).

## Effects of DHT on Various Other Biological Processes and Tissues

T and its metabolites DHT and estradiol have well-known effects on nongonadal tissues including, but not limited to, the prostate. Determining the relative importance of DHT in mediating the androgen effects of T in humans relies predominantly on investigating the impact of DHT suppression, because the provision of exogenous DHT results in compensatory reductions in endogenous T (and estradiol) due to negative feedback in the brain and pituitary gland and likely in peripheral tissues as well. Moreover, the relatively higher potency of DHT compared with T as a result of tighter/longer binding to AR complicates the interpretation of dose- vs androgen-specific or tissue-selective effects when the effects of exogenous DHT are evaluated. Inhibition of SRD5A results in very modest increases, if any, in circulating T (124–126), thus providing a reasonable context in which to evaluate the requirement for DHT in maintaining peripheral androgen effects. Of note, the broader SRD5A antagonist, dutasteride, is particularly effective for these types of investigations, as it is a potent inhibitor of both SRD5A type I and type II, whereas finasteride is a less potent inhibitor of SRD5A type I (127).

### Erythropoiesis

Androgens, but not estradiol, increase erythropoiesis and have some clinical utility in the treatment of mild anemias associated with long-term hypogonadism as recently confirmed in older hypogonadal men (128). DHT can serve to stimulate erythropoiesis when given in supraphysiologic dosing, despite suppressing endogenous T and estradiol (E) (54), but it is not required for exogenous T to exert these effects (129–131). Furthermore, endogenous DHT, despite its androgenic potency, is not necessary for maintenance of normal hematocrit and hemoglobin in healthy men (131, 132). Recent data point to suppression of hepcidin and increased erythropoietin production as the mechanisms whereby T increases erythropoiesis and iron incorporation into red blood cells (133, 134). Normal levels of circulating DHT are not required for suppression of hepcidin (135).

In the Page *et al.* (55) study, there was no change in hematocrit after subjects were treated with a DHT gel preparation for 1 month. In the Idan *et al.* study, eight subjects in the DHT arm were discontinued due to polycythemia over the 24-month treatment period (54). Compared with most other TRT studies where intervention (*e.g.*, dose adjustment, phlebotomy, or discontinuation) does not occur unless a hematocrit >54% has been confirmed, a conservative hematocrit limit of >50% was used by Idan *et al*. None of these subjects with elevated hematocrit were symptomatic or required intervention. Interestingly, these men all had high-normal hemoglobin levels at baseline. The magnitude of response to DHT [group mean (± standard error) hematocrit: 47.1 ± 1.3 in DHT vs 43.4 ± 0 in the placebo group; *P* < 0.001] was the same in men who did or did not become polycythemic. In contrast with these findings, Jockenhovel *et al.* (136) compared four TRT preparations (including oral TU treatment of 55 hypogonadal men) and found a positive correlation existed with T and hematocrit and hemoglobin levels, but not DHT.

Studies of T combined with a 5AR-I (*e.g.*, finasteride or dutasteride) also fail to show an appreciable effect of DHT on erythrocytosis (129–131). In these studies, the effect of T alone on hematocrit was compared with T plus a 5AR-I over periods of exposure ranging from 20 weeks to 36 months. In men treated for 36 months, T therapy alone (200 mg T enanthate (TE) intramuscularly every 2 weeks) resulted in an increase in hematocrit from a mean baseline of 42.9% to 48.6% at month 36. Men treated with T plus finasteride (5 mg, daily) also experienced an increase in hematocrit from 43.2% at baseline to 47.4%. Despite a significant reduction in circulating levels of DHT (50% below baseline), there was no effect of lower DHT levels on the magnitude of erythrocytosis when compared with men treated with T alone.

Clearly, the DHT levels observed in the literature in response to TRT are many fold less than has been observed when DHT gel has been used in clinical trials. Consequently, it is probable that chronic exposure to very high DHT will increase hematocrit in some men. It is noteworthy that all subjects in the Idan *et al*. study, who discontinued due to polycythemia, had the highest baseline hemoglobin levels (albeit still in the normal range), a reflection, perhaps, of polymorphisms that predispose some men to DHT-mediated effects on hematocrit when treated with DHT gel. Such a polymorphism in the erythropoietin gene that influences hematocrit levels in normal blood donors has been described (137).

Overall, the available data do not support DHT as the principal driver of changes in hematocrit observed in response to the various TRT routes of administration. The erythrocytosis observed in response to TRT seems predominantly due to a direct inhibitory action of T (independent of DHT) on hepcidin transcription/expression and to increased iron incorporation in red blood cells (133, 135). However, it also has been shown that androgens may directly affect erythropoietin concentrations via stimulation of bone marrow hematopoietic stem cells (138, 139), a pathway that involves AR-mediated induction of IGF-1 (140). A direct role of DHT in this pathway has not been investigated.

### Lipids

In contrast to T therapy which has been associated with decreases in HDL cholesterol that are dependent, in part, on route of administration and circulating levels of T, transdermally administered DHT [even at levels in the range of 700 ng/dL (24.27 nmol/L) for up to 2 years] has not been associated with detrimental shifts in total cholesterol, HDL and LDL cholesterol, or triglycerides (52, 54). A similar response has been observed in men treated with T in combination with a 5AR-I (finasteride or dutasteride) that resulted in suppression of DHT to levels far below the normal range. In these studies, there were no differences in lipid response between men treated with T or those treated with T plus finasteride or dutasteride (129, 130). Orally administered TU has been associated with a modest increase in DHT and a significant drop (roughly 30%) in HDL (141, 68). But it seems unlikely that this lowering of HDL is predominantly due to DHT but instead to T-mediated effects in liver secondary to portal absorption of enterocytic-derived T from the enzymatic hydrolysis of TU by nonspecific esterases (142).

“DHT-gel treatment did not significantly increase total, central or peripheral prostate volumes.”

### Skin

Skin possesses all of the requisite steroidogenic capabilities to ensure local homeostatic control of steroid hormones, suggesting an important paracrine role for T, DHT, and estradiol within the skin, the function of which is poorly understood (143). Likewise, skin contains metabolizing pathways (*e.g.*, glucuronidation; sulphation) that inactivate DHT (144). Consequently, localized mechanisms in skin maintain concentrations of DHT that are not meaningfully influenced by circulating DHT levels, probably due to the fact that the DHT concentration gradient favors secretion into blood (143, 145). In men, androgen levels are highest in the scrotal skin followed by pubic skin and then thigh skin, a pattern paralleled by 5*α*-reductase and low 3*α*-reductase activity (and thus tissue DHT) (146). In women, skin DHT concentrations are highest in the labia majora and clitoris followed by pubic skin and then thigh skin (147).

Absorption of DHT (as is also true for T) across the skin is a passive process that follows Fick’s law (148, 149). Accordingly, concentrations of DHT in skin to which DHT has been applied are extremely high during the period when DHT is absorbed into the bloodstream. A single transdermal DHT product is available in a handful of countries, and it is this formulation that has been used in clinical trials. Studies of topical DHT in hypogonadal or eugonadal men have not reported adverse effects on skin aside from mild irritation due to the high alcohol content, despite its direct application and sustained supraphysiological levels of DHT for up to 24 months (51, 52, 54). These results are consistent with those from shorter-term transdermal DHT studies (60, 103).

Male human skin and hair express an abundance of SRD5A type I in sebaceous glands, hair follicles, sweat glands, and the epidermis, whereas SRD5A type II is expressed in genital keratinocytes and hair follicles (150). The physiologic role of DHT in the skin is unclear, but it is hypothesized that sex steroids may influence the immune function of skin and locally influence inflammatory processes (143). Androgens clearly play a role in the pathogenesis of acne vulgaris, likely through increased sebum production, and may impact cutaneous wound healing (151); however, a specific role for DHT in many of these processes has not been demonstrated. An exception to this may be DHT-induced upregulation of inflammatory cytokines (*e.g.*, IL-1, IL-6, and tumor necrosis factor-*α*) in acne (152). Studies of agents that reduce levels of DHT in skin clearly support a role for DHT in the development of male androgenic alopecia (MAA) (male pattern baldness). This is inferred, in part, by the effectiveness of 5AR-Is in suppressing the progression of MAA and the observations that castrated men and men with SRD5A deficiency do not develop baldness (153). However, the effectiveness of SRD5A therapy likely resides at the level of the hair follicle (*i.e.*, lowered follicular concentrations of DHT) and not a reduction of circulating DHT because this has not been shown to correlate with MAA. Support for this conclusion is also found in a study of men exposed to exceptionally high levels of DHT in response to daily application of a DHT gel preparation for 24 months. DHT was not associated with acne, MAA, or other androgen-associated skin pathology (54). Instead, the most important factor in the pathogenesis of MAA is a genetic predisposition for AR polymorphisms [*e.g.*, synonymous nucleotide polymorphism in exon 1 (rs6152) of the AR] (154, 155). In addition, differences in AR concentrations and steroid-converting enzymes in the hair follicle also appear to be play a significant role in MAA (156).

### Body composition

Exogenous T increases lean body mass and bone mineral density (BMD) while decreasing fat mass. Furthermore, endogenous T is required for maintenance of these tissues (157, 158). A recent controlled study in older hypogonadal men demonstrated that T therapy for 12 months in older men with low T significantly increased volumetric BMD and estimated bone strength, more in trabecular than peripheral bone and more in the spine than hip (159). Both T and DHT have been shown to inhibit preadipocyte proliferation and adipocyte differentiation and to stimulate lipolysis, thus providing mechanistic evidence for the reduction in fat mass observed in hypogonadal men undergoing T replacement therapy (160, 161). In addition, DHT (like T) has been shown to have dose-dependent inhibitory effects on lipoprotein lipase activity in human adipose tissue explants (162).

#### Impact of exogenous DHT therapy on body composition

Mårin *et al*. (163, 164) compared the effects of transdermally applied T and DHT to a transdermal placebo treatment on changes in body composition and triglyceride uptake and release from adipose tissue in middle-aged (mean age, 58 years) eugonadal men with abdominal obesity who were treated daily with these androgens for 9 months. In response to DHT, circulating mean levels of DHT increased to 223 ng/dL (7.68 nmol/L) at the end of the treatment whereas mean T levels declined to hypogonadal levels. Elevated DHT was not associated with statistically significant changes from baseline for body weight, body mass index, waist or hip circumference, lean body mass, total fat mass, and subcutaneous fat mass. There was a modest but statistically significant increase in visceral fat of 0.5 kg. DHT treatment was without effect on triglyceride uptake in abdominal and femoral subcutaneous adipose tissue and in lipoprotein lipase activity in abdominal fat.

A more recent 2-year, placebo-controlled study of DHT therapy in healthy, middle-aged men (mean, 61 years) has been reported by Idan *et al.* (54). Fat and lean mass were measured by dual-energy x-ray absorptiometry and by bioelectrical impedance. In response to sustained supraphysiological mean serum DHT concentrations exceeding 800 ng/dL (27.54 nmol/L), lean mass increased and fat mass decreased by 1.0 to 1.5 kg, respectively, compared with little or no change in the placebo cohort. These changes, albeit modest in magnitude, are consistent with general androgen action in fat and muscle tissue. And whereas these results differ from those of Mårin *et al*. (163, 164) described previously, daily DHT exposures in this study were significantly higher (3.5×) and for a much longer period of time (24 vs 9 months). From these studies, we conclude that supraphysiological levels of DHT in men have a modest effect on body composition to decrease fat mass and increase lean mass. And this may also hold true in women. Cote *et al.* (165) has challenged the assumption that high androgen levels in women are associated with abdominal obesity based on their study of 60 women that found plasma DHT was negatively correlated with total adiposity as well as computed tomography assessments of abdominal obesity (both subcutaneous and visceral). A significant negative association was also observed between plasma DHT and omental adipocyte diameter. These findings are consistent with those of Gruber *et al.* (166), who reported that transdermal DHT treatment of postmenopausal women significantly reduced total body and abdominal fat when assessed by dual x-ray absorptiometry.

Given the androgenic potency of DHT, particularly in adipose tissue where the equilibrium of several key processes (*e.g.*, adipocyte differentiation, lipid accumulation, and lipolysis) are directly influenced by androgens, it is tempting to reason that changes in circulating DHT may affect these processes. But this appears not to be the case because, in both muscle and fat cells (as is the case in prostate tissue), intracellular androgen concentrations (*e.g.*, DHEA, T, and DHT) are mediated by biochemical control mechanisms that tightly control local androgen levels by efficiently metabolizing excess androgen to inactive metabolites (167, 168). DHT is extensively metabolized in fat tissue to 5*α*-androstane-3*α*, 17*β*-diol prior to glucuronidation by two key AKR1C isozymes, namely, AKR1C1 and AKRIC2 (also known as 20*α*-hydroxysteroid dehydrogenase and 3*α*-HSD, respectively) (160). Hence, despite the fact that fat tissue may serve as a steroid reservoir, it also is a site of very active steroid metabolism that functions to create a local equilibrium for steroid action (169).

**Figure 6.**
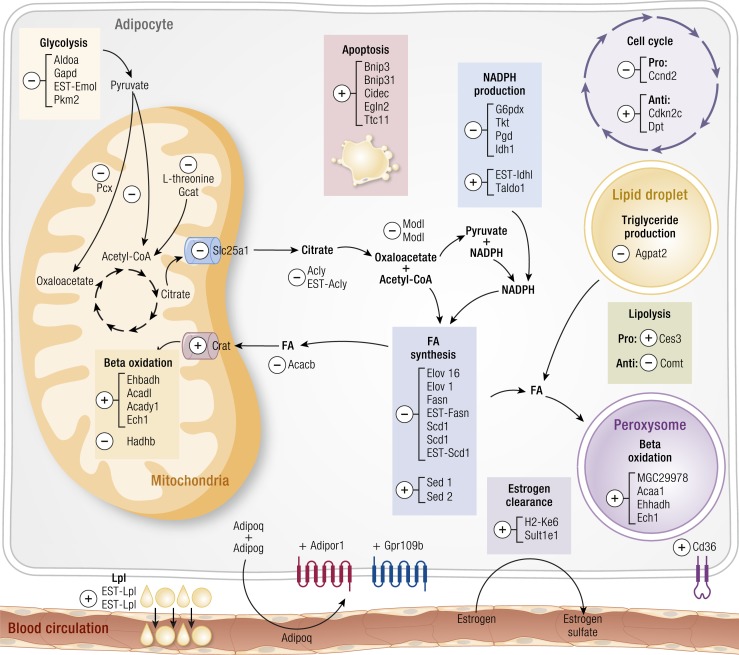
Overview of the effects of DHT on various pathways in adipose tissues. **+** indicates upregulation, and – indicates downregulation of grouped genes. Collectively, DHT modulates several pathways involved in energy metabolism and promotes lipid use by multiple mechanisms. See original reference for list of full gene names. Redrawn from Bolduc *et al.* (170). acetyl-coA, acetyl co-enzyme A; FA, fatty acid; Lpl, lipoprotein lipase; NADPH, nicotinamide adenine dinucleotide phosphate.

Long-term transcriptonomic effects of DHT exposure on various genes in murine adipose tissue have been reported, but these effects have not been evaluated in humans (170). As summarized in Fig. 6, DHT modulated several key pathways of energy metabolism, including stimulation of lipid disposal and downregulation of lipogenesis. DHT also promoted several transcripts associated with apoptosis of adipocytes while simultaneously suppressing cell cycle progression (170). Whether these DHT-induced effects also occur in human adipose tissue remains an area for future study, but the consistent action of DHT to stimulate lipid use and suppress its synthesis in mammalian tissue suggests that some of these actions may occur in humans.

#### Effects of 5*α*-reductase inhibition therapy and BMD on body composition

Tenover and colleagues performed the first and longest study demonstrating that normal serum levels of DHT were not required to maintain androgen effects on body composition and BMD when older, hypogonadal men were treated with either placebo, TE injections, or TE plus finasteride for 3 years (129, 171). T therapy was associated with significant gains in lean body mass and BMD, along with increases in physical function. Fat mass also decreased in both groups receiving T. Notably, finasteride had no impact on these positive changes. Similar results were reported more recently in a 1-year study that used higher doses of TE alone or in combination with finasteride (131). Dutasteride in combination with T also did not impact the dose-response effects of T on BMD, body composition, and leg strength (130). Although DHT is not required for these positive effects of T replacement, supraphysiologic DHT levels do provide for sufficient androgenicity to build lean body mass and reduce fat mass (54, 172). But exogenous DHT may also reduce circulating T and estradiol, thus leading to a reduction in BMD as has been reported for the spine in middle-aged and older men (54). These results are in keeping with the known importance of estrogens in male bone health (173). Fewer long-term, placebo-controlled studies have examined the impact of 5AR-Is outside of the context of T replacement in men on these anabolic end points, but in a 12-month trial in healthy young men, neither dutasteride nor finasteride (that significantly lowered serum DHT) altered body composition nor BMD (131, 132). Moreover, a case control study of men treated with finasteride for BPH found no increased risk of hip fracture (174). In concert, these findings demonstrate that endogenous DHT is not required for maintenance of body composition and BMD when T levels are within the normal physiologic range. However, estradiol appears necessary to maximize bone health in men.

### Metabolic syndrome and type 2 diabetes

Low levels of T and DHT have been reported in men with metabolic syndrome and/or type 2 diabetes, and T deficiency has been associated with obesity (particularly visceral), insulin resistance, and dyslipidemia. In addition, low T is a risk factor for the development of diabetes and associated cardiovascular sequel (175–177). In a randomized, controlled trial, TRT has been associated with beneficial effects on insulin resistance, total and LDL cholesterol, lipoprotein, and sexual health in diabetic hypogonadal men (178). However, until the benefits and risks of TRT in this patient population have been more fully clarified, there remains controversy about widespread TRT in the clinical management of hypogonadal men with metabolic syndrome or type 2 diabetes (179). Interestingly, although there is a paucity of data regarding what role, if any, DHT plays in the either metabolic syndrome or type 2 diabetes, relatively new research findings suggest DHT may play multiple beneficial roles. First, as noted previously, DHT therapy has been associated with decreased fat mass and increased lean body mass. Next, DHT stimulates lipid use and suppresses its synthesis by various intracellular pathways (Fig. 6). Third, under exercise conditions that would be expected to improve glycemic control in type 2 diabetic patients, muscle glucose metabolism has been shown to increase in parallel with concentrations of DHT in type 2 diabetic rats, effects that were inhibited by a SRD5A inhibitor (180). Fourth, resistance training in older men induced expression of key steroidogenic and AR proteins in muscle and restored muscle concentrations of T and DHT to those observed in young men (167, 181). Collectively, these actions may aid glycemic control. Fifth, in a 12-month, randomized, controlled trial, moderate-intensity aerobic exercise in middle- to older-aged men significantly increased circulating levels of DHT and SHBG (182). And finally, Joyce *et al.* (183) recently published findings from a study in which the relationships between T, DHT, and SHBG with incident risk of diabetes in older men were evaluated in the Cardiovascular Health Study. Androgens in this study were assayed by LC-MS/MS. Low concentrations of DHT were strongly associated with higher insulin resistance and higher risk of diabetes, and this negative association remained even after adjusting for covariates known to contribute to diabetes risk and modifiers such as binding proteins (SHBG). These finding are consistent with those of Mather *et al.* (184). More clinical research is needed to better understand how DHT modifies diabetes risk and whether, for example, therapies that increase DHT are beneficial in reducing this risk or improving clinical outcomes of importance in diabetes.

### Sexual function

Perhaps the most controversial area with regards to the nonprostate-related effects of DHT in male health is in the area of sexual function, particularly with respect to the reversibility, or lack thereof, of sexual side effects that are attributed to the 5AR-Is. Multiple large studies have reported sexual dysfunction in men with BPH taking finasteride or dutasteride, including three large, placebo-controlled studies (185–187). Overall, use of 5AR-Is is associated with a 5% to 9% prevalence of new-onset erectile dysfunction and decreased libido (188). These data strongly point to a role for endogenous DHT in the maintenance of normal sexual and ejaculatory function and sexual desire. And, in fact, this has been shown to be true in healthy young men where serum DHT concentration was revealed to be the sole independent predictor of orgasm frequency, a surrogate for overall male sexual function (189).

The impact of reductions in serum DHT in younger men, who may be using 5AR-Is for MAA, is more controversial (190). In particular, some retrospective analyses and case series have suggested that sexual side effects in younger men taking 5AR-Is are irreversible (190); however, it should be noted that such analyses may suffer from recall bias, and all lack a placebo control, so the findings should be treated with caution until appropriately designed trials are conducted. Of note is one placebo-controlled trial in healthy young men that revealed small changes in sexual function and semen parameters (but not fertility) with both finasteride and dutasteride over 1 year (124, 132). But all of these changes reverted to baseline 6 months after drug cessation. The role of estrogens in sexual desire and erectile function in men was recently highlighted in an elegant study from Finkelstein *et al.* (191). Studies of exogenous DHT are largely consistent with these findings and show that DHT maintains or improves sexual function, including a minor role (secondary to T) in maintenance of normal erectile function (52, 172, 192). However, possibly through decreases in serum estradiol concentrations, elevated DHT may not help to maintain sexual desire (172). Notably, inclusion of dutasteride with T replacement did not alter sexual function in older men with low T (130). In summary, endogenous DHT appears to play a role in the maintenance of normal sexual function, including libido, in younger and older men. We have not identified any literature to suggest modest or even substantial increases in circulating DHT have a negative impact on male sexual function.

A recent study of men who reported persistent sexual symptoms after finasteride use for hair loss found no evidence of androgen deficiency, persistent inhibition of peripheral SRD5A activity, or diminished peripheral androgen action (193).

### 5*α*-reductase deficiency

5*α*-reductase deficiency (5ARD) is an autosomal recessive disorder in 46,XY males that results in the inability to convert T to DHT due to a fault in the critical gene that codes for SRD5A2 (194). Because DHT is obligatory for normal masculinization of external genitalia, the disorder is typically characterized by striking ambiguity of the external genitalia (195). Biochemical characteristics of 5ARD principally include increased T and decreased DHT levels, increased T:DHT ratio, decreased plasma 3*α*-androstanediol glucuronide, and abnormal ratios of urinary 5*α*- to 5*β*-steroid metabolites (194). Treatment of 5ARD with transdermal DHT preparations that increased circulating DHT to adult levels has been reported (196, 197). Parenteral administration of DHT heptanoate (DHT-hp) at 4- to 6-week intervals that resulted in a sustained elevation of plasma DHT levels has also been used in the clinical management of 5ARD (198). However, only one of these DHT preparations is commercially available (*i.e.*, DHT gel) in a small number of countries, excluding the United States, and none have been evaluated for 5ARD in longer-term efficacy and safety studies.

### Gynecomastia

Elevations in DHT have not been associated with gynecomastia, as would be expected given a role for estradiol that cannot be directly formed from DHT. For this very reason, transdermal DHT (199) and a parenteral DHT prodrug DHT-heptanoate (DHT-hp) has been explored as a treatment of spontaneous gynecomastia in men or adolescent boys (198, 200). Intramuscular injection of 200 to 400 mg DHT-hp every 2 to 4 weeks for 16 weeks was associated with a 67% to 78% decrease in breast size in adolescent boys with gynecomastia; no regrowth was observed for up 15 months post treatment. Circulating DHT (as measured by RIA after Celite chromatography) increased by week 16 of therapy to a mean concentration of 278 ng/dL (9.6 nmol/L). Over this period, there was a progressive decrease in T secondary to a DHT-mediated decrease in LH at the hypothalamic level (201) and in estradiol secondary to suppression of T. The corresponding DHT/T ratio was 8.2 (compared with 0.14 at baseline). DHT-hp was well tolerated. There was no change in testicular volume. DHT-hp therapy resulted in modest increases in body and facial hair and in body weight. Two cases of increased acne were reported. There was no change in liver or renal function. Additional, and appreciably larger, studies are needed to more fully determine the clinical utility and safety of DHT-hp in the nonsurgical treatment of gynecomastia.

### Cognition

The cognitive effects of androgens have been difficult to ascertain, and there are few studies that have specifically focused on the role, if any, of DHT on cognition in men or women. Recent cross-sectional data from Australia have revealed that declines in serum T, DHT, calculated free T, and estrone (but not estradiol) over time in men >70 years of age were associated with poorer cognitive function (202). The mechanism for this relationship is unknown, but it is possible that low circulating androgen levels lead to detrimental effects on cognitive function. Others have reported that treatment of men with mild cognitive impairment and low T may benefit from T replacement therapy and that aromatization of T to estradiol is critical for improvement of verbal but not spatial memory (203, 204). Alternatively, cognitive decline may be a direct cause of a reduced androgen status, a hypothesis that is contradicted by the fact that cognitive decline is not a hallmark of longstanding male hypogonadism (205). In rodents, T but not DHT improves working memory in aging male rats (206), whereas in female rats, T and DHT improved different aspects of cognition. These findings raise the possibility of some androgen selectivity, although a potency or aromatase effect could not be ruled out (207). There is significant expression of SRD5A in the human brain, particularly of type I, making it likely that there are local, tissue-specific effects of DHT on cognition and/or mood that may be difficult to discern in the context of systemic hormonal alterations, despite the fact that lipophilic steroid hormones like T and DHT can cross the blood-brain barrier (208, 209). Expression of SRD5A type 1 mRNA in the human brain has primarily been in the temporal and frontal lobes (208). Although finasteride and dutasteride both can cross the blood-brain barrier (210, 211), the predominant expression of SRD5A type 1 in the brain makes dutasteride a better agent for studying the effects of DHT inhibition in humans on cognition and mood. Nonetheless, we have been unable to identify any long-term studies of the impact of dutasteride on cognition in men.

Replacement of normal elderly men with low T levels without cognitive deficits with T or T plus finasteride failed to demonstrate significant effects on cognition over 3 years compared with placebo, although there was a small improvement in attention with T and in verbal memory with the addition of finasteride. Gray *et al.* found a dose-response relationship between T dose and visual-spatial cognition over 20 weeks in healthy older men (212), consistent with improvements noted in other studies of exogenous T (213). One small study has compared the effects of exogenous T vs DHT in the correction of hypogonadism in otherwise healthy older men, noting that T improved verbal memory, whereas DHT (which lowered T and E concentrations) resulted in improved spatial memory, implying some tissue selectivity of the various sex steroids (214). A recent study on the effect of DHT on synaptic plasticity of the hippocampus in male senescence-accelerated mouse prone 8 (SAMP8) mice (a good model of cognitive decline due to its similarities to Alzheimer’s disease) found that DHT treatment promoted expression of synaptic plasticity markers [namely, cAMP-response element binding protein (CREB), postsynaptic density protein 95 (PSD95), synaptophysin (SYN), and developmentally regulated brain protein (Drebrin)], positively modified synaptic structure, and significantly delayed cognitive impairment (215). In light of the fact that low androgen levels have been identified in men (and women) with dementia and Alzheimer’s disease (216, 217), further large, placebo-controlled studies are of value to determine whether DHT has any effect in maintaining cognitive health.

### Telomere length

Telomere shortening has been well documented as a biochemical marker of cell aging (218), and a potential role for T in attenuating this process via stimulation of telomerase activity has been observed in human ovarian cells (219) and in animal models of aplastic anemia (220). The impact of DHT on telomere length has recently been compared with T and estradiol in leukocytes harvested from 980 middle-aged men (221). DHT and estradiol correlated with increased leukocyte telomere length. In men with polymorphisms of the aromatase gene that were associated with reduced estradiol, telomere length was shortened. More research is needed to determine whether T therapy, via its partial metabolism to DHT and estradiol, might preserve health in older men via outcome-based studies. However, it is intriguing to speculate that the increase in all-cause and cancer-specific mortality observed in men with low T and DHT (222–224) may, in part, reflect reduced actions of DHT and estradiol to preserve telomere length. There is an association in some studies between short telomere length and prostate cancer, but the effects of DHT on leukocyte telomere length may not reflect what occurs in prostate tissue. However, in prostate biopsies from men in the Prostate Cancer Prevention Trial, shorter telomere length was associated with higher odds of prostate cancer (225). Because the concentration of DHT is very high in the prostate, one may hypothesize that if DHT stimulates telomere lengthening in prostate, it may paradoxically play a protective role in some cells.

## DHT in Women

The principal circulating androgens in women based on both production rates and serum levels are the sulfated form of DHEA (DHEA-S) > DHEA > androstenedione > T > DHT (226). As is the case in men, T and DHT are bound primarily to SHBG and albumin. The very low concentrations of T [about 40 ng/dL (1.4 nmol/L)] and DHT [about 10 ng/dL (0.34 nmol/L)] have led to controversy over the accuracy of their measurements, particularly by RIA techniques (227). Rothman *et al.* used LC-MS/MS to assess changes in serum androgen and estrogen levels in pre- and postmenopausal women (228). This study revealed mean serum DHT concentrations in healthy pre- and postmenopausal women of about 9 ng/dL (0.3 nmol/L) and 3 ng/dL (0.1 nmol/L), respectively. But unlike T and free T that peak at midcycle, DHT levels did not change across the menstrual cycle. Compared with T, women produce one-tenth the quantity of DHT each day (226).

In females, the role of DHT remains unclear. Excess circulating androgen is a key feature of polycystic ovary syndrome (PCOS), and hyperandrogenism [either biochemical or clinical (*e.g.*, acne and/or hirsutism)] is a component in all but one of the specific phenotypes associated with PCOS (229). Although the measurement of circulating T and androstenedione is common in the clinical evaluation of PCOS and may be useful for predicting metabolic risk, DHT is not routinely measured in this patient population (230). An exception to this is a study Munzker *et al.* (231) in which total T and DHT, calculated free T and DHT, and the ratio of T/DHT were measured by LC-MS/MS in a cohort of women (n = 275) with PCOS and in a matched control group. Only total T, calculated free T and DHT (secondary to a significant decrease in SHBG), androstenedione, and calculated free DHT were elevated in the PCOS group; no difference in total DHT was observed. However, a strong link between the T/DHT ratio and adverse hormonal, anthropometric, and metabolic parameters was observed in women with PCOS, leading the authors to propose the T/DHT ratio as a new biomarker for PCOS. Substantially larger trials are needed to corroborate these findings.

Hirsutism, a common symptom of PCOS, is another example where circulating levels of DHT (or its 3*α*- and 3*β*-androstanediol metabolites) do not appear to play a significant role compared with intracellular concentrations (232–234). In hirsute women, it is well established that increased type I 5-AR in the hair follicle acts on T to produce high local concentrations of DHT that transforms vellus hair (nonpigmented, soft, and short) to terminal hair (pigmented, course, and long) in androgenic-sensitive areas of the skin (235). DHT is metabolized to 3*α*-androstanediol glucuronide within the hair follicle with subsequent release into the circulation, suggesting that this metabolite is a good marker of peripheral androgen production in women with idiopathic hirsutism (226). These studies were performed before more accurate and precise measurements of DHT were possible with LC-MS/MS.

There are several factors at play in the development of acne in women, but primary among these is increased local androgen levels that lead to excessive production of sebum. Unlike circulating levels of T and DHEA-S that have been shown to be associated with acne development in women, the role of DHT is unclear. Elevated levels of serum DHT are uncommon (236). Of probable greater importance is the local formation of DHT from T and androsterone in the pilosebaceous unit. Hence, androsterone glucuronide has been recommended as the best serum biochemical marker of acne in hyperandrogenic women (237).

Just as androgens play a role in the development of hirsutism, they also can contribute to hair loss in women. But there appears to be no evidence that circulating levels of DHT affect this process. Cela *et al.* assessed DHT levels in women with androgenic alopecia and compared them to a normal control group (238). There was no difference in circulating DHT between groups. Vierhapper *et al.* determined production rates of T and DHT in young women (23 to 40 years old) using the stable isotope dilution technique and mass spectrometry (239). In the presence of normal metabolic clearance rates, production rates of T were increased. Metabolic clearance of DHT was below normal, but DHT production rates were within or below the normal range. In contrast to male pattern baldness, female pattern hair loss was characterized by increased production rates of T, but not of DHT. For this reason, assessing circulating levels of DHT in women with androgenic alopecia or female pattern baldness would appear to be of little diagnostic value in clinical practice.

We conclude this section with a brief analysis of DHT in pregnancy-induced hypertension (PIH). Alterations of T and androstenedione profiles have been implicated in the pathophysiology of PIH/pre-eclampsia (240–242). To further explore this possible association, Jirecek *et al.* (243) investigated serum concentrations of T, DHT, androstenedione, and DHEA-S measured by enzyme-linked immunosorbent assay in women with PIH (n = 40) and normotensive pregnant women (n = 40). Median serum concentrations of androstenedione and T were significantly elevated in women with PIH vs controls. In contrast, there was no significant change in serum levels of DHT or DHEA-S in women with PIH compared with levels in the control group. Although reassuring with respect to DHT, this study was small and thus confirmation in a larger trial would be beneficial.

## Does the DHT/T Ratio Provide Any Additional Information Beyond Serum DHT Measurements?

Clinically, DHT/T ratios are useful to rule out 5ARD or excess androgen synthesis through the backdoor pathway in female virilization disorders (*e.g.*, PCOS). A recent study by Munzker *et al.* (231) has provided evidence for a strong link between a high T/DHT (*i.e.*, a low DHT/T) ratio and an adverse metabolic phenotype in PCOS patients. However, beyond instances in which the determination of the DHT/T ratio will guide a clinical decision, we believe that undue attention has been dedicated to the calculation of a DHT/T ratio based on circulating androgen levels. Moreover, DHT often is not measured or reported in studies of TRT. It is not clear from our literature review why this ratio has been and continues to be used in pharmacokinetic studies of androgen therapy or as a safety monitoring parameter of TRT because it does not provide any additional information beyond serum DHT levels which, if elevated, are alone sufficient for correlation with clinical safety end points. This is not true in the case of T and estradiol, in which a ratio has a biologic rationale given the opposing action of these hormones in most tissues. Because T and DHT act on the same receptor and are tightly controlled at the intracellular level in androgen-sensitive tissues, changes in DHT/T ratio based on circulating levels of these androgens do not provide information on which any clinical decision would or should be made in the context of TRT for the clinical management of hypogonadal men.

A potential alternative approach to the DHT/T ratio may be to use the sum of T plus DHT, the two most potent naturally occurring androgens, as a means to assess total (or net) clinical androgen status, particularly in research studies in which an exogenous androgen (*e.g.*, DHT) is administered to eugonadal men. In such cases, endogenous production of T will be suppressed but the total androgen status can be gauged by measurement of T plus DHT (103). In studies of TRT, we see no benefit to this approach compared with the measurement of T and DHT alone. Handelsman *et al.* (48) describe a method for assessing net androgen effects by defining a new “serum androgen” value as the sum of serum T plus 5 times the serum DHT concentration [*i.e.*, serum androgen = T + (5 × DHT)]. The rationale behind this approach reflects the higher potency of DHT vs T based on DHT affinity for the AR and its slower dissociation rate compared with T. However, as described earlier, there are numerous factors at the level of AR binding beyond AR affinity and dissociation that determine which androgen ultimately interacts with the AR and the resultant integrated downstream transcription events. Nonetheless, this approach is interesting and worthy of further use as a potentially more comprehensive method to characterize androgen status in some studies, particularly epidemiology studies in which the effects of DHT and T differ or in studies in which use of overall androgen status may provide new insights.

Regardless of the method chosen to assess total androgen status, both hormones should be carefully measured by the same validated method (*e.g.*, LC-MS/MS) and the total value considered in the context of, for example, the eugonadal T plus DHT range for the particular analytical laboratory used. Only if the sum of T plus DHT were to fall above the upper limit of normal for the combined T plus DHT androgen concentration would there be a theoretical safety concern. However, we are not aware of any study of TRT where T plus DHT or T plus 5 times the serum DHT concentration either prospectively or retrospectively (*e.g.*, meta-analyses) has been correlated with safety outcomes.

## Serum DHT and DHT/T Ratios Observed in Response to T Therapy in Men With Low T

### Transdermal T preparations

Numerous transdermal preparations of T are available for the clinical management of hypogonadal men and include a nonscrotal T patch, T gel preparations of various T concentrations, and a T solution applied to the axilla. Historically, the first transdermal T product was a T patch applied daily to the scrotum (244). Although no longer available, its use was associated with elevated DHT levels presumably due to high levels of 5*α*-reductase in scrotal skin (244). Atkinson *et al.* (73, 245) followed a cohort of hypogonadal men (N = 25) treated continuously with the scrotal T patch for 8 years. The approximate mean serum concentration of DHT (as measured by RIA; see “Analytical Methods for DHT Quantification”) and the DHT/T ratio during this time was 175 ng/dL (6.03 nmol/L) and 0.42, respectively. Atkinson *et al.* noted that reports of prostate disease (carcinoma or hyperplasia) with the scrotal T patch were infrequent. Of 681 men treated with this T patch for 6 months to >9 years, only 14 (2.0%) cases of BPH and 4 (0.58%) cases of prostate cancer were identified. Given the age of the study population and duration of follow-up, this frequency was not substantially different than the general population treated with TRT. In addition, changes in prostate volume in response to this form of T therapy were modest and tended not to progress after about 18 months.

T gel/solution preparations have largely supplanted use of T patch products. These products are applied daily to the skin whereupon the solubilized T is absorbed. At steady state, these products all yield an average serum T concentration that is roughly the midnormal range (70, 71). As expected due to relatively high 5*α*-reductase activity in skin, the serum DHT and DHT/T ratios increased in response to these products. For example, serum DHT levels tripled from baseline to about 130 ng/dL on day 90 after application of 5 g of a 1% T gel and were approximately fivefold higher in men who applied 10 g of the gel. The DHT/T ratios increased to approximately 0.25 to 0.30 in response to T gel, a response also observed after 42 months of therapy (71). Lower DHT/T ratios of 0.147 to 0.172 have been reported in studies of a 1.62% T gel, but these remain about twofold higher than baseline (246, 247).

T gels have not been associated with a significant number of prostate-related adverse events even though serum DHT levels modestly increase in response to treatment. Across several studies, mean PSA, urine flow, IPSS scores, and prostate size did not change significantly One-year exposure to AndroGel 1.62% did not yield significant changes to various inflammatory biomarkers (*i.e.*, hs-CRP, IL-6, IL-10, and VCAM) nor to circulating fibrinogen levels. Despite this, it should be noted that long-term outcome studies of T gels have not been conducted to quantify risk of prostate disease, most notably, cancer.

A 2% T solution for underarm application (*i.e.*, to the axillae) is also available for TRT. Daily application of this product to the axilla normalizes serum T concentration along with a concomitant increase in serum DHT (248). Mean steady-state serum DHT concentrations rose from a baseline of 18 to 98 ng/dL (0.62 to 3.37 nmol/L) over 120 days of therapy. Similarly, the mean steady-state DHT/T ratio increased to 0.26 (72). This form of TRT has not been associated with an increased incidence of prostate-related adverse effect, but long-term outcomes data are lacking.

### Parenteral T preparations

The effects of parenterally administered TE on circulating DHT levels have been studied by Lakshman *et al.* (9). As expected, serum DHT increased in a dose-dependent manner over the range of weekly TE doses administered to men with GnRH agonist-induced hypogonadism. For example, following a 125-mg T dose (roughly equivalent to the weekly dose used in the clinical management of hypogonadal men), mean serum DHT (measured by LC-MS/MS) increased from a baseline of approximately 20 ng/dL (0.69 nmol/L) to about 70 ng/dL (2.41 nmol/L). In response to a 300-mg TE dose (a supraphysiologic dose when the dosing interval is 2 to 3 weeks), sustained serum DHT concentrations of about 110 ng/dL (3.79 nmol/L) were observed. There was little difference in response between younger (mean age, 26 years) and older men (mean age, 66 years). In contrast to serum DHT response, DHT/T ratio decreased in a dose-dependent manner but also did not differ between older and younger men. A slightly higher serum DHT response [mean of about 75 ng/dL (2.6 nmol/L) with peak of 116 ng/dL (2.5 to 4 nmol/L)] has been observed following a single dose of a 140-mg T dose as either TE or T cypionate, but T and DHT were measured by an older RIA method and thus may overstate actual androgen concentrations (249). Of note is that the DHT response was identical for either T prodrug.

The effects in hypogonadal men treated with a long-acting, intramuscular formulations of TU on circulating levels of DHT and the DHT/T ratio have been reported by Wang *et al.* (TU formulation approved for use in the United States) (77) and Schubert *et al.* (TU formulation widely approved in other regions, for example, the European Union and Asia) (250). TU was administered by Wang *et al.* at a dose of 750 mg in 3 mL of castor oil at baseline, week 4, and every 10 weeks thereafter for 84 weeks. Mean serum DHT (measured by LC-MS/MS) increased gradually over time from a baseline of about 17 ng/dL (0.59 nmol/L) to about 25 ng/dL (0.86 nmol/L) and 30 ng/dL (1.03 nmol/L) at weeks 14 and 64, respectively. The serum DHT/T ratio (0.075) did not change appreciably over the course of TU therapy. Serum PSA increased with TU treatment, and two subjects were diagnosed with prostate cancer. This incidence was considered by the investigators to be consistent with other TRT preparations dosed for a similar length of time. As with other parenteral TRT products, outcome studies to prospectively assess TRT risk on prostate remain elusive.

Schubert *et al.* evaluated the efficacy and safety of 1000 mg parenteral TU at 6-week intervals for the first three doses (*i.e.*, loading dose) then every 9 weeks for 30 weeks. Serum DHT (as measured by LC-MS/MS) at week 30 increased from a baseline of about 8.7 to 29 ng/dL (0.3 to 1 nmol/L) but always remained within the normal range [9.3 to 72.5 ng/dL (0.32 to 2.5 nmol/L)]. No adverse effects of this treatment regimen on prostate have been observed (251, 252).

### Subcutaneous T implants (pellets)

The effects of subcutaneous T pellet implants on DHT have been evaluated in a single-dose, open-label, nonrandomized pharmacokinetic study (76). In brief, six T pellets (each containing 200 mg of T) were implanted in the subdermal fat layer of lower abdomen in 14 hypogonadal men. Serum DHT (as measured by RIA after oxidative destruction of T to remove potential cross-reactivity of T with DHT) was significantly elevated from day 21 to day 105 and correlated with a significant rise in serum T. Peak DHT concentrations of approximately 290 ng/dL (10 nmol/L) were observed at about day 60. Over the course of 200 days post implantation, the average serum DHT concentration was approximately 116 ng/dL (4 nmol/L).

### Nasal T preparation

Treatment of hypogonadal men with a 4.5% T nasal gel resulted in mean DHT and DHT/T values after 90 days that were in the normal physiologic ranges [33.2 to 40.1 ng/dL (1.14 to 1.38 nmol/L) and <0.1, respectively] (Table 3) (69). These results are generally at the lower end of the range when compared with other approved TRT products. There were no reports of increased PSA levels in subjects receiving the BID dosing, and 6.1% of subjects receiving TID dosing showed increased PSA levels. Long-term safety data for this product have not been reported.

### Oral TU preparations

An oral form of T replacement therapy that utilizes TU in an oil formulation was originally developed in the 1970s (253). Although never approved as a TRT in the United States, it remains on the market in over 80 countries across the world. A unique aspect of TU is its absorption exclusively via the intestinal lymphatics whereupon T is liberated via the action of nonspecific esterases (142). The typical dose for this product ranges from 80 to 200 mg/d of TU, which is roughly equivalent to 50 to 125 mg/d of T. A major disadvantage of this TU formulation is that the serum T response tends to be relatively low, as evidenced by mean T levels below the eugonadal range or only sporadically within normal limits (253, 254). In addition, this formulation must be administered with food containing some fat to ensure adequate bioavailability. Serum T, DHT, and DHT/T ratios observed after administration of this oral TU formulation in hypogonadal men are summarized in Table 4 (46, 75, 255–257). In four studies in which sufficient data were reported, the average DHT/T ratios in response to therapeutic doses of oral TU ranged from 0.34 to 0.52. These DHT/T ratios are generally higher than ratios reported for most T replacement products and may reflect, in part, use of an immunoassay for analyzing DHT concentrations (68). Long-term exposure of hypogonadal men to oral TU, with the resultant elevations in the DHT/T ratios, has not resulted in toxicity, particularly related to the prostate gland. Gooren has provided the most comprehensive chronic safety summary for oral TU (75). Thirty-five hypogonadal men (aged 15 to 62 years) were administered oral TU (80 to 200 mg/d) for 10 years. Mean DHT concentrations (measured by RIA) increased modestly above the upper normal limit of 73 ng/dL (2.1 nmol/L) to about 96 ng/dL (3.31 nmol/L) over the period that the men were followed. The DHT/T ratio averaged 0.54 over the 10-year period compared with a reference range of 0.08 to 0.125. Despite these higher serum DHT and DHT:T ratios, long-term exposure to oral TU was not associated with any adverse effects on prostate as assessed by digital rectal exam, urine flow studies, and PSA. None of the subjects in the study developed prostate cancer.

**Table 4.** **Summary of Mean Serum T and DHT Concentrations and DHT/T Ratios From Studies of a Non–Self-Emulsifying-Delivery Oral TU Formulation*^a^***

Oral TU Dose	N	Duration	T (ng/dL) [nmol/L]	DHT (ng/dL) [nmol/L]	DHT/T	Androgen Assay Method
80 mg BID (with food) (46)	5	Several months	232 ± 147 (SD) [8.04 ± 5.10]	93 ± 42.3 [3.20 ± 1.46]	0.40	RIA
80 mg BID (with food) (255)	14 (female)	Single day	316 ± 111*^c^* [10.9 ± 3.48]	165 ± 75*^c^* [5.68 ± 2.58]	0.52	GCMS
80 mg QD (with food) (256)	24 (female)	Single day	175 ± 37*^c^* [6.07 ± 1.28]	59 ± 50*^c^* [2.03 ± 1.72]	0.34	LC-MS/MS
80–200 mg/d (with food)*^b^* (75)	35	12 mo	155 ± 55 [5.37 ± 1.91]	101 ± 35 [3.48 ± 1.21]	0.65	RIA
		36 mo	173 ± 58 [5.99 ± 2.01]	99 ± 38 [3.41 ± 1.31]	0.57	
		60 mo	176 ± 52 [6.1 ± 1.8]	93 ± 52 [3.2 ± 1.79]	0.53	
		72 mo	170 ± 49 [5.89 ± 1.7]	96 ± 49 [3.31 ± 1.69]	0.56	
		84 mo	187 ± 55 [6.48 ± 1.91]	102 ± 49 [3.51 ± 1.69]	0.55	
		108 mo	193 ± 52 [6.69 ± 1.8]	93 ± 46 [3.20 ± 1.58]	0.48	
		120 mo	187 ± 40 [6.48 ± 1.39]	90 ± 41 [3.10 ± 1.41]	0.48	

Abbreviations: GCMS, gas chromatography – mass spectrometry; QD, once daily; SD, standard deviation.

^*a*^ The oral TU formulation used in these studies was available under the tradename Andriol®.

^*b*^ Values for T and DHT are mean (rounded to nearest whole number) ± SD. DHT/T is derived from the T/DHT, which was reported in the noted reference.

^*c*^ Estimated from provided area under the curve (AUC) data according to the general formula: *C*_ss_ = AUC/*T*, where *C*_ss_ is steady-state concentration, AUC is area under the dose concentration curve, and *T* is dosing interval.

Newer formulations of oral TU for TRT are in development. One such formulation (CLR-610) for which clinical data has been published contains TU in a self-emulsifying drug delivery system (258). Inherent in this formulation are excipients that foster the solubility and bioavailability of TU even when taken in a fasted state. Nonetheless, optimal TU absorption occurs when administered with food containing a typical level of fat. The pharmacokinetics of CLR-610 have been evaluated in trials reported by Yin *et al.* (68). After 28 days of oral TU administration at a dose of 316 mg TU, BID (equivalent to 200 mg T, BID), the average serum DHT levels increased from a baseline of 21 to 110 ng/dL (0.7 to 3.8 nmol/L), and the serum DHT/T ratio increased from 0.09 to approximately 0.3. The DHT and DHT/T ratio reference range reported by Yin *et al.* was approximately 14 to 77 ng/dL (0.5 to 2.7 nmol/L) and 0.04 to 0.11, respectively, as measured by LC-MS/MS.

Why oral TU tends to increase DHT more than parenteral forms of TU probably relates to several factors associated with the absorption of TU. As noted previously, TU enters the circulation only by intestinal lymphatic absorption. In the enterocyte, TU can be hydrolyzed to T by nonspecific esterases whereupon it can be further metabolized to DHT by 5*α*-reductase. DHT formed in intestinal tissue is rapidly metabolized to inactive glucuronides in gut or, if absorbed into the portal circulation, in liver tissue (6, 46) and thus contributes appreciably little, if any, to the circulating DHT pool. But TU can also be converted by enterocytic 5*α*-reductase to dihydrotestosterone undecanoate (DHTU), which then may be absorbed lymphatically. Action by nonspecific esterases on DHTU yields DHT, and it is this pathway that probably accounts for the modest elevations in DHT observed in response to oral TU preparations (142). Transient high concentrations of TU and DHTU occur after oral TU, and these may also impact circulating DHT (68). However, the impact of this must be considered small given modest increases in DHT after oral TU at a time of robust levels of TU and DHTU. Notably, TU and DHTU do not effectively bind to the AR due to an absence of a hydroxyl group at the C-17 position on T and steric hindrance created by the presence of the undecanyl group at this same position (21). Furthermore, TU and DHTU are rapidly metabolized and do not accumulate in the circulation after oral TU (68), nor are they sequestered in tissue (259).

## Key Conclusions and Recommendations for Future Clinical Research

Circulating levels of DHT in response to TRT do not correlate with those found in androgen-sensitive tissue (*e.g.*, prostate, adipose, muscle) due to local regulatory mechanisms that tightly control intracellular androgen homeostasis. Observations from numerous clinical studies are consistent with current knowledge that androgen-sensitive tissues can self-regulate tissue DHT levels by downregulating its synthesis and upregulating metabolism during DHT excess or, conversely, upregulating synthesis and downregulating metabolism under conditions of T or DHT deprivation. We are reminded of Horton’s admonition some 25 years ago when he concluded that blood levels of DHT provide only a hint of tissue levels and that DHT should be regarded as a paracrine hormone formed and acting primarily within target tissues (39).

The modest increases observed in serum DHT and in the DHT/T ratio observed after TRT are unlikely to be a cause of clinical concern, particularly when viewed in the context of changes observed in these parameters for currently marketed T replacement products and those under development for which DHT data are available. There is no sound current clinical evidence to indicate that elevated DHT concentrations (either short-lived peaks or sustained supraphysiological levels) are associated with risk beyond that known for androgens (most notably, T), including adverse effects on prostate.

Epidemiological data, especially from the placebo arm of the REDUCE trial, have failed to show any relationship between circulating levels of DHT and risk for prostate cancer. Although well-controlled, long-term studies designed to specifically examine the effects of androgen exposure on risk for prostate need to be conducted, the current clinical database is relatively reassuring that circulating levels of androgens (or changes in such) apparently do not play as pivotal a role as once thought in the development of prostate disease.

Robust epidemiologic or clinical trial evidence of a deleterious DHT effect on CVD is lacking. To the contrary, there is some evidence that DHT therapy in men with CVD may improve clinical status, a finding that needs confirmation. We acknowledge recently published data from a longitudinal database of older normal men (*i.e.*, not hypogonadal) that indicated an association between serum DHT and incident CV disease and mortality. At the same time, others have reported that higher DHT levels in older men were associated with decreased all-cause mortality and reduced ischemic heart disease mortality. Studies of this nature suffer from their observational nature. Additional exploration in prospective, placebo-controlled intervention studies of TRT with CVD as the primary end point is needed to resolve the long-term effects of androgens on CVD risks.

DHT does not play a substantive role in body composition compared with T under normal conditions. Thus, elevated levels of DHT in response to TRT are unlikely to appreciably impact lean or fat mass. Nonetheless, data from animals suggest a role for DHT in adipose tissue that inhibits biochemical pathways involved in lipid synthesis and promotes several transcripts associated with apoptosis of adipocytes. Whether these DHT-induced effects also occur in human adipose tissue remains an area for future study.

There is very limited data available regarding DHT and effects on cognition. Further research is needed, particularly in light of animal data where DHT positively modified synaptic structure and significantly delayed cognitive impairment in a well-regarded animal model for Alzheimer’s disease.

Recent data indicating that higher levels of DHT were inversely associated with insulin resistance and risk of diabetes merit further mechanistic investigation to understand whether this action is separate from that of T.

In summary, we have reviewed evidence that slightly to moderately elevated DHT concentrations or an elevated DHT/T ratio during androgen therapy (most notably, TRT) are unlikely to pose either a higher risk or a unique risk compared with T. We acknowledge that the available published data are limited by the lack of large, well-controlled studies of long duration that are sufficiently powered to expose subtle safety signals. Nonetheless, the preponderance of available clinical data leads to the conclusion that modest elevations in circulating levels of DHT in response to androgen therapy should not be of concern in clinical practice.

## Abbreviations:

3*α*-HSD: 3*α*-17*β*-hydroxysteroid dehydrogenase
5ARD: 5*α*-reductase deficiency
5AR-I: 5*α*-reductase inhibitor
ADT: androgen deprivation therapy
AR: androgen receptor
BMD: bone mineral density
BPH: benign prostate hypertrophy
CAD: coronary artery disease
cAMP: cyclic adenosine monophosphate
CI: confidence interval
CVD: cardiovascular disease
DHEA: dihydroepiandrosterone
DHEA-S: sulfated form of dihydroepiandrosterone
DHT: dihydrotestosterone
DHT-hp: dihydrotestosterone heptanoate
EPC: endothelial progenitor cell
HCD: high-cholesterol diet
HDL: high-density lipoprotein
ICAM: intercellular adhesion molecule
LC-MS/MS: liquid chromatography tandem-mass spectrometry
LDL: low-density lipoprotein
MAA: male androgenic alopecia
mRNA: messenger RNA
PCOS: polycystic ovary syndrome
PIH: pregnancy-induced hypertension
PSA: prostate-specific antigen
R_SHBG_: sex hormone–binding globulin receptor
SHBG: sex hormone–binding globulin
T: testosterone
TE: T enanthate
TRT: testosterone replacement therapy
TU: testosterone undecanoate
UGT: UDP-glucuronyltransferase
VCAM: vascular cell adhesion molecule.
